# The interaction between the immune system and the autonomic nervous system in the pathophysiology of major depressive disorder: a scoping review

**DOI:** 10.1016/j.bbih.2026.101334

**Published:** 2026-08-22

**Authors:** Emma Saillart, Katleen Jacobs, Stephan Claes, Elfi Vergaelen

**Affiliations:** a Mind-Body Research, Department of Neurosciences, KU Leuven, Leuven, Belgium; b University Psychiatric Center KU Leuven, University Hospital Leuven, Leuven, Belgium

**Keywords:** Major depressive disorder, Autonomic nervous system, Immune system, Inflammation, Heart rate variability

## Abstract

Major depressive disorder (MDD) has been associated with both immune dysregulation and autonomic nervous system (ANS) dysfunction, and growing evidence suggests that bidirectional interactions between these systems may contribute to depression pathophysiology. However, the existing evidence has not yet been comprehensively synthesized, limiting understanding of the ANS–immune axis in MDD.

This scoping review aimed to map the literature investigating interactions between the ANS and immune system in MDD, identify methods assessing this relationship, and explore implications for stratification.

Following PRISMA-ScR and Joanna Briggs Institute guidelines, a systematic search of 12 databases and trial registers was conducted through January 2026. Eligible studies included human studies examining both ANS functioning and immune markers in MDD. Data were synthesized across three ANS domains: catecholaminergic signaling, cardiac autonomic measures, and vagus nerve functioning.

Twenty-five studies published between 1989 and 2025 were included. Studies on catecholamine–immune interactions reported both positive associations and null findings between catecholamines and inflammatory markers, while in vitro studies showed both suppressive and enhancing immune effects. Several studies investigating heart rate variability (HRV) have reported inverse associations between inflammatory markers and vagally mediated HRV indices, although many of these studies lacked covariate adjustment. Studies investigating vagal function and vagus nerve stimulation reported immunomodulatory effects, but these varied according to stimulation modality, duration, and participant stratification.

Current evidence suggests that altered ANS functioning may be associated with immune dysregulation in MDD, but findings remain heterogeneous. Larger longitudinal studies with standardized methods are needed to clarify these ANS–immune interactions.

## Highlights

• This review mapped ANS–immune interactions in MDD.
• Lower vagal HRV was linked to higher inflammation.
• ANS–immune findings in MDD were highly heterogeneous.
• Methodological variability limited comparison across studies.
• Stratification may improve understanding of ANS–immune dysfunction in MDD.

## 1 Introduction

Over the past few decades, major depressive disorder (MDD) has become one of the most widespread mental disorders and the leading cause of disease-related disability worldwide, posing a major global public health challenge (Liu et al., 2020a). However, progress in understanding its underlying mechanisms remains limited because individuals who share the same diagnosis show considerable interindividual heterogeneity in their clinical presentation, suggesting that multiple biological pathways may contribute to the disorder (Monroe and Anderson, 2015; Goldberg, 2011). Identifying more homogeneous subgroups within MDD could enhance our understanding of the disorder's specific etiological and biological mechanisms and support the development of more effective personalized diagnostic and therapeutic approaches (Kas et al., 2025; Beijers et al., 2019).

One of the biological mechanisms that has been extensively investigated in MDD is the immune system. Meta-analytic evidence indicates that individuals with MDD show altered peripheral inflammatory markers (Osimo et al., 2020) and immune cell counts (Foley et al., 2023), as well as increased levels of pro-inflammatory cytokines in cerebrospinal fluid (CSF) and increased activation of microglia (Enache et al., 2019). Genetic and transcriptomic studies further indicate that shared genetic variants and gene-expression pathways contribute to both immune activation and depression (Hepgul et al., 2016; Mostafavi et al., 2014; Bull et al., 2009), and increased expression of inflammatory genes has been linked to poorer response to conventional antidepressants (Barnes et al., 2017). Experimental research also supports a causal contribution of inflammation to MDD: administering cytokines or inflammatory challenges induces depressive symptoms (McNutt et al., 2012; Grigoleit et al., 2011), and anti-inflammatory treatments have been shown to alleviate depressive symptoms, particularly in patients with elevated inflammation (Du et al., 2024).

Despite strong evidence for immune alterations in MDD, not all patients demonstrate these changes. Approximately 30% of individuals diagnosed with MDD appear to exhibit an immune-related subtype of depression. This subgroup is often identified by elevated levels of high-sensitivity C-reactive protein (hsCRP), an acute-phase protein produced by the liver during inflammation (Osimo et al., 2019). Patients with this immune-related subtype frequently show greater antidepressant treatment resistance (Chamberlain et al., 2019), a higher burden of symptoms (Foley et al., 2021) and a higher prevalence of cardiometabolic comorbidities, including obesity and metabolic syndrome (Rethorst et al., 2014).

In addition to inflammation, MDD is also associated with autonomic nervous system (ANS) dysfunction. Numerous studies have demonstrated that MDD is linked to an increased risk for the development and progression of cardiovascular diseases, with ANS dysregulation considered one of the key pathways underlying this relationship (Kemp et al., 2012; Tobaldini et al., 2020). For example, heart rate variability (HRV), representing the variation in time between successive heartbeats and reflecting the interplay between the sympathetic and parasympathetic branches of the ANS, serves as a reliable biomarker of ANS functioning (Shaffer and Ginsberg, 2017). Individuals with MDD consistently exhibit reduced HRV and elevated heart rate (HR) (Koch et al., 2019; Schiweck et al., 2019), with greater alterations observed in severe forms of MDD (Kemp et al., 2010). Alterations in autonomic functioning in MDD have also been observed using other physiological measures, including blunted resting skin conductance and stress-induced electrodermal activity responses (Kim et al., 2018), as well as blunted and less stable skin temperature rhythms (Lorenz et al., 2019).

**Fig. 1:**
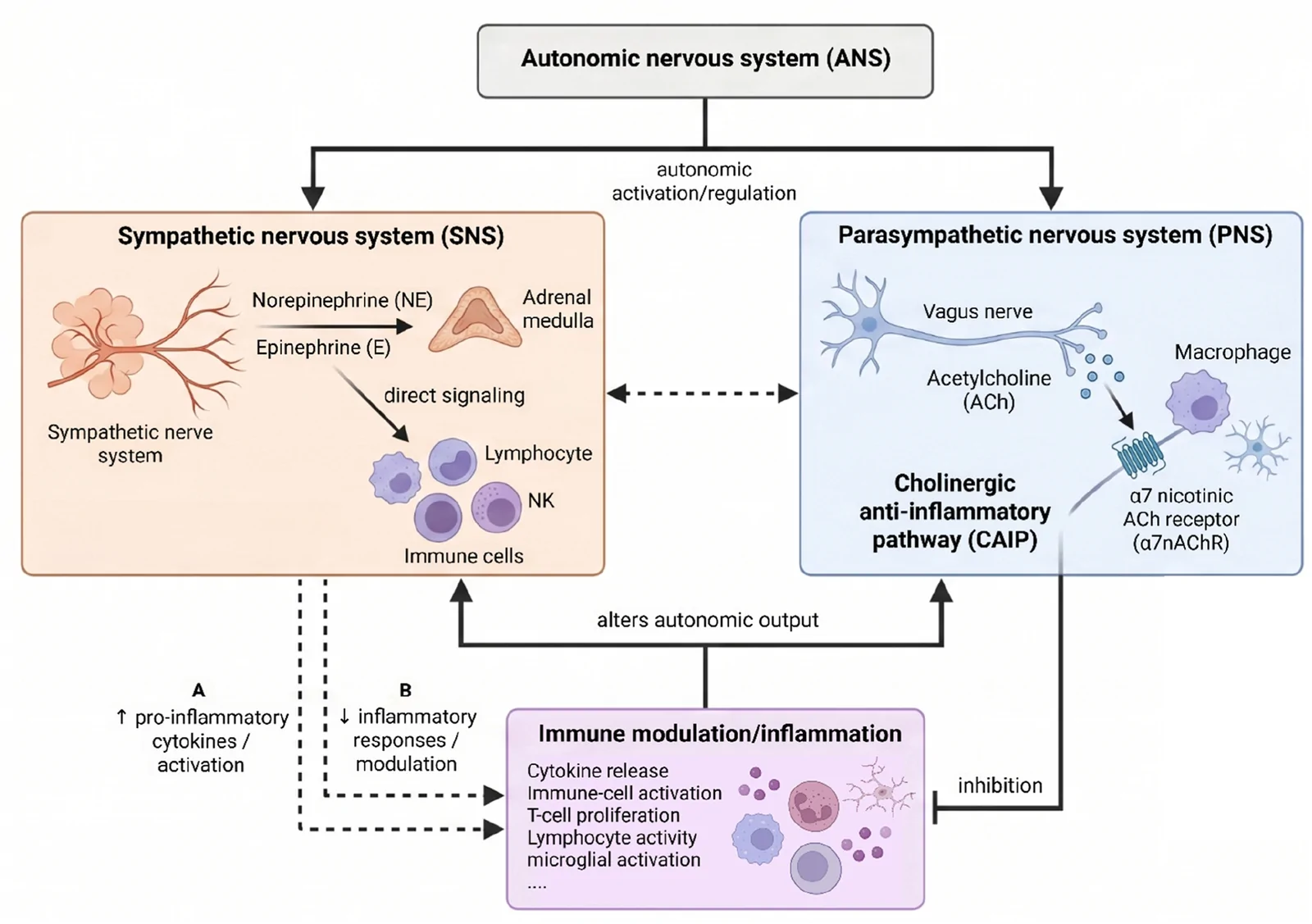
**Bidirectional interactions between the autonomic nervous system and the immune system.** The ANS regulates immune functioning through both sympathetic and parasympathetic pathways. The sympathetic nervous system modulates immune activity via catecholamine release, including norepinephrine and epinephrine, which act on adrenergic receptors on immune cells and can exert both pro-inflammatory (A) and anti-inflammatory or immunomodulatory (B) effects depending on physiological context. In parallel, the parasympathetic nervous system, primarily via the vagus nerve, suppresses inflammatory activity through the cholinergic anti-inflammatory pathway in which acetylcholine activates α7 nicotinic acetylcholine receptors on immune cells, thereby inhibiting pro-inflammatory cytokine release. Immune activation can in turn alter autonomic functioning, illustrating the bidirectional relationship between the ANS and the immune system. Created with BioRender.com.

Accumulating evidence has established a bidirectional relationship between the ANS and the immune system. For example, sympathetic mechanisms can influence immune response through sympathetic innervation of lymphoid organs and the activation of adrenergic receptors on immune cells (Kenney and Ganta, 2014). Subsequently, the parasympathetic system, via the vagus nerve, can suppress inflammation through efferent vagal fibers that inhibit pro-inflammatory cytokine release via nicotinic acetylcholine receptors, also known as the cholinergic anti-inflammatory pathway (CAIP) (Tracey, 2002). Moreover, studies introducing immune challenges have demonstrated that immune activation can, in turn, alter ANS function (Takase et al., 2005; Kox et al., 2011). To provide an overview of these interacting mechanisms, Fig. 1 summarizes the proposed bidirectional pathways linking ANS dysregulation and immune functioning.

Given this established bidirectional relationship, dysregulation of the ANS–immune axis may play a significant role in the pathophysiology of MDD. In this scoping review, we therefore aim: (1) to map the existing clinical literature investigating the interaction between the immune system and the ANS in MDD, including the extent to which these interactions differ from healthy controls and relate to depressive symptoms; (2) to identify the methodological approaches used to study ANS and immune functioning; (3) to explore the potential of ANS–immune measures as candidate biomarkers for patient stratification. Given that research directly examining the interplay between the ANS and the immune system in MDD remains limited and fragmented, a scoping review is warranted to clarify the current state of knowledge and identify key research gaps.

## 2 Methods

### 2.1 Protocol and registration

This scoping review was conducted in line with Joanna Briggs Institute Reviewers’ Manual and Preferred Reporting Items for Systematic Reviews and Meta-analyses: Extension for Scoping review (PRISMA-ScR) (Tricco et al., 2018). A scoping review methodology was selected, rather than a systematic review, to enable a comprehensive synthesis and mapping of existing evidence within a field characterized by conceptual complexity, heterogeneity, and limited prior review (Pham et al., 2014).

The review process was guided by the methodological framework initially proposed by Arksey and O'Malley (Arksey and O'Malley, 2005), incorporating subsequent refinements by Levac et al. (2010) and Peters et al. (2015). This framework comprises five iterative stages: (1) identifying the research question; (2) identifying relevant studies; (3) selecting studies; (4) charting the data; and (5) collating, summarizing, and reporting the results. The final protocol was registered prospectively in the Open Science Framework (https://osf.io/qwaev).

### 2.2 Eligibility criteria

The review included studies enrolling participants with a current diagnosis of MDD (any age/sex, inpatient and outpatient) as defined by standardized diagnostic criteria (e.g. Diagnostic and Statistical Manual of Mental Disorders (DSM) and International Classification of Diseases (ICD)). Studies primarily focusing on psychiatric disorders other than MDD were excluded. However, studies reporting comorbid psychiatric conditions were eligible provided that MDD was the primary diagnosis under investigation. We included empirical studies of any design with primary human data, as well as registered clinical trial reports and preprints, provided that they reported primary data. Theoretical papers, opinion pieces, reviews, meta-analyses, case reports, and case series were excluded. Eligible studies investigated immune system function (e.g., cytokine concentrations, hsCRP) and ANS function (e.g., HRV, norepinephrine), and MDD severity when available, which was assessed through validated self-report scales or clinician-rated questionnaires. Only studies published in English were considered.

### 2.3 Search strategy and selection of sources of evidence

The initial data search was carried out across 12 information sources and was completed on 6 March 2025. We searched: PubMed (including MEDLINE, via NCBI), Scopus (via scopus.com), Embase (via embase.com), CINAHL (via EBSCOhost), Web of Science Core Collection (via webofscience.com), CENTRAL (via the Cochrane Library), Psychology Database (via ProQuest), APA PsycArticles (via ProQuest), Europe PubMed Central preprints (medRxiv, bioRxiv), ClinicalTrials.gov, and the WHO International Clinical Trials Registry Platform (ICTRP). We additionally performed backward and forward citation tracking to identify further relevant records. To ensure currency of the evidence base, the search was updated on 14 January 2026, using the same search strategy and eligibility criteria.

The search strategy targeted the key concepts “major depressive disorder” AND “immune system” AND “autonomic nervous system” and combined controlled vocabulary (e.g., MeSH, Emtree) with free‐text terms. The complete, reproducible search strings for each source are documented in Supplementary File 1. All search results were exported and de-duplicated in EndNote according to pre-specified rules.

Study selection proceeded in two stages. First, two independent reviewers (ES and KJ) screened titles and abstracts of all de-duplicated records against the predefined eligibility criteria using Rayyan in blinded mode (Ouzzani et al., 2016). Second, the same reviewers independently assessed the full texts of potentially eligible studies based on the inclusion criteria.

### 2.4 Data extraction and analysis

A standardized data-charting form was developed in Microsoft® Excel for data extraction. Both reviewers (ES and KJ) independently charted data and verified each other's entries for completeness and accuracy. Discrepancies were resolved by consensus, and adjudication was used when necessary**.**

Data were abstracted based on characteristics of the articles (e.g., type of article or study), population characteristics (e.g., type of MDD patients), intervention characteristics and outcomes (e.g. immune outcomes, ANS outcomes).

## 3 Results

### 3.1 Selection and characteristics of sources of evidence

**Fig. 2:**
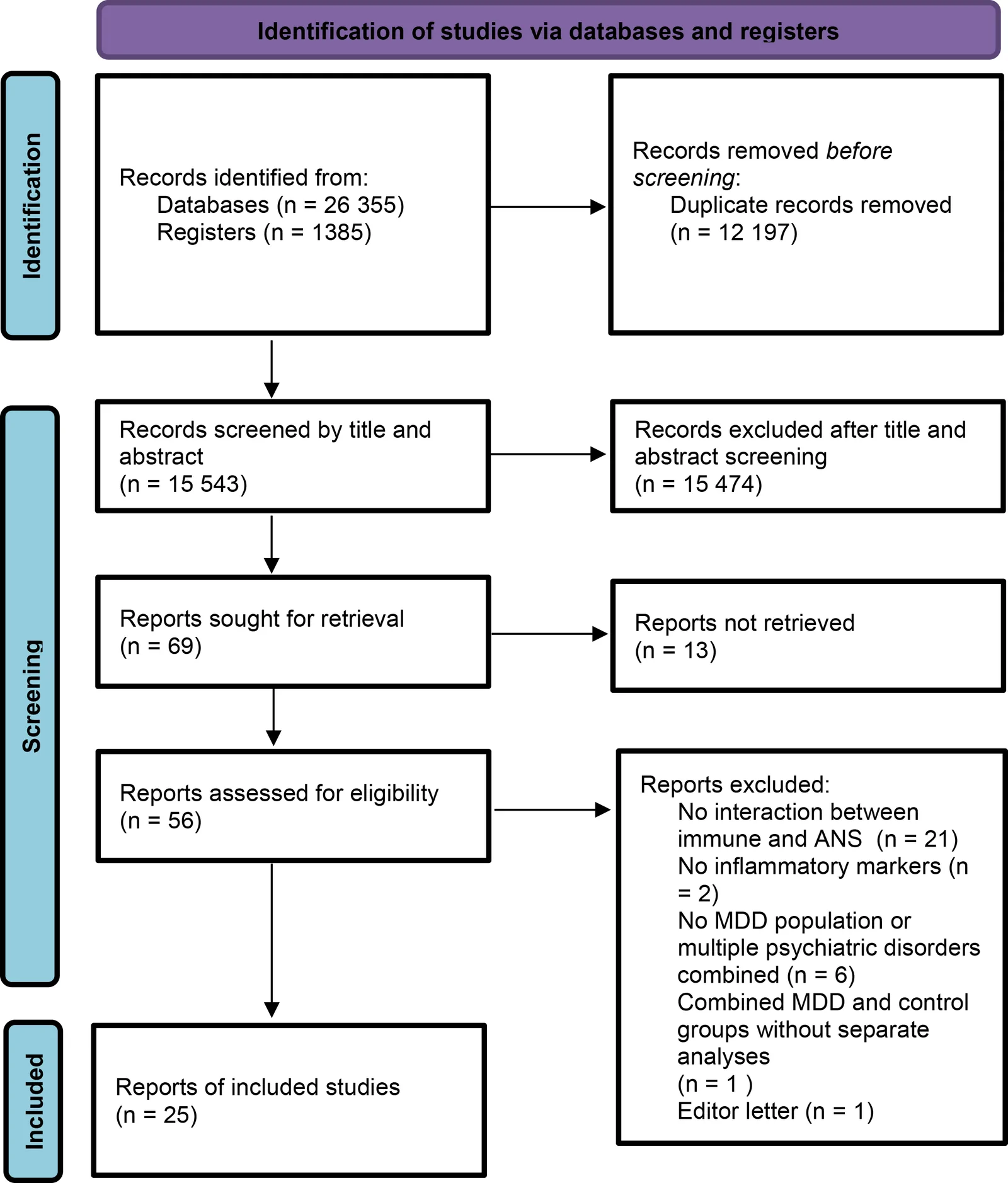
PRISMA flow diagram.

The overall selection process through the different screening phases is summarized in a PRISMA flow diagram (Fig. 2). The literature search across multiple databases yielded a total of 27 713 records. After removal of duplicates in EndNote, 15 543 unique records remained and were screened by title and abstract for eligibility according to the predefined inclusion and exclusion criteria. Of these, 15 474 records were excluded, leaving 69 reports eligible for full-text retrieval. Thirteen reports could not be retrieved; therefore, 56 articles underwent full-text screening, of which 31 were excluded for the specific reasons detailed in the PRISMA flow diagram provided in Fig. 2. In total, 25 studies met the inclusion criteria and were included in the final synthesis.

The search yielded 25 studies published between 1989 and 2025. The included articles comprised quantitative studies of various designs, including 13 cross-sectional, 9 experimental, and 3 intervention studies. For each study, the following information was extracted and summarized in Table 1, Table 2, Table 3: first author (year), group characteristics, medication status, MDD severity assessment, proportion of female participants (% of total sample), mean age (years), body mass index (BMI), immune outcomes, ANS outcomes, and intervention or measurement method.

**Table 1:** Characteristics of included studies investigating SNS and immune markers in MDD.

First author (year)	Group	AD status	MDD scoring	Gender (%F)	Age M (SD)	BMI M (SD)	Immune outcomes	SNS markers	Biological matrix	Measurement method
Maes et al. (1989)	19 md 18 MD - M 11 MD + M	20 drug-naive pre-admission 26 drug-naive post-admission	HDRS	57.9% md 61.1% MDD- M 63.6% MDD + M	39.5 (16.6) md 42.6 (11.9) MDD- M 47.1 (12.0) MDD + M	/	Lymphocyte stimulation by mitogens	MHPG	Urine	HPLC
Irwin et al. (1991)	19 MDD 19 HC	16% drug-naive 15-21 days 84% drug-naive > 3 months	HDRS-24	0% (all men)	48.8 (12.2) MDD 48.1 (12.5) HC	/	NK-cell cytotoxicity activity	E NE	Plasma	COMT radioenzymatic assay E, NE RIA NPY
								NPY		
Hickie et al. (1995)	28 MDD	100% drug-naive	HDRS-21	42.9%	49.3 (13.9)	/	CMI with DTH skin responses	NE	Urine	GC-MS
								MHPG		
Ravindran et al. (1996)	18 MDD 31 dysthymic 23 HC	100% drug-naive	HAM-D MADRS CGI	50% MDD 58.1% Dys 60.9% HC	41.22 (2.59) Dys 37.55 (3.72) MDD 35.13 (2.49) HC	/	Lymphocyte subsets (CD3, CD4, CD8, CD19, CD16/56)	NE E	Plasma	HPLC
Anisman et al. (1999)	14 dep 31 Atyp-dep 14 Dysthymic 15 Atyp-Dys 27 HC	100% drug-naive	HAM-D MADRS BDI	66.6% MDD 55.2% Dys 59.3% HC	41.43 (5.51) Dep 39.87 (2.97) Atyp-Dep 41.84 (4.06) Dys 39.31 (2.98) Atyp-Dys 35.82 (2.52) HC	/	Lymphocyte produced IL-1 βand IL-2	E NE	Plasma	HPLC
Irwin et al. (2003)	14 MDD 31 HC	14.3% drug- naive 20-21 days	HDRS-21	0% (all men)	41.2 (10.0) MDD 44.4 (11.4) HC	/	(IL-2 stimulated) NK cytotoxicity activity	E NE	Plasma	COMT radioenzymatic assay
Mata et al. (2005)	35 MDD 29 HC	100% drug-naive	HAM-D	82.9% MDD 75.9% HC	36.68 (1.69) MDD 31.52 (1.08) HC	/	Peripheral lymphocyte noradrenergic turnover/transporter function	NE MHPG	Plasma	HPLC
Weinstein et al. (2010)	14 MDD 14 HC	86% drug-naive	HDRS BDI-II	50% MDD 50% HC	39.3 (5.6) MDD 41.7 (9.6) HC	25.2 (4.4) MDD 26.0 (3.8) HC	CRP, IL-6, TNF-α	E NE	Plasma	HPLC
Lopes et al. (2012)	22 MDD - CMT 16 MDD + CMT 16 HC	100% AD monotherapy (SSRI or TCA)	BDI	100%	39.73 (10.12) MDD - CMT 41.06 (6.46) MDD + CMT 37.07 (5.64) HC	26.65 (2.27) MDD - CMT 26.18 (2.25) MDD + MCT 25.37 (2.77) HC	T-cells (PBMCs), IL-2, IL-4, IL-6, IL-10, TNF- α, IFN- gamma	E	Plasma	HPLC
Futtrup et al. (2018)	108 MDD 44 HC	100% drug-naive	HAMD-17	64.80% MDD 68.20% HC	41.6 (11.5) MDD 38.6 (12.5) HC	26.6 (6.0) MDD 26.6 (6.2) HC	hsCRP, IL-6	NE	Plasma	ELISA
Yoshimura et al. (2019)	148 MDD 40 HC	100% drug-naive	HAM-D17	56.10% MDD 42.5% HC	49.5 (12.1) MDD 44.0 (10.5) HC	/	IL-6	MHPG HVA	Plasma	HPLC

**Note.** BMI = body mass index; MDD = major depressive disorder; HC = healthy controls; MD = major depression; MD − M = major depression without melancholia; MD + M = major depression with melancholia; md = minor depression; atyp-dep = atypical depression; atyp-dys = atypical dysthymia; AD = antidepressant; CMT = childhood maltreatment; BDI = Beck Depression Inventory; BDI-II = Beck Depression Inventory II; HAM-D/HAMD-17/HDRS/HDRS-17/HDRS-21/HDRS-24 = Hamilton Depression Rating Scale (17-, 21-, or 24-item versions); MADRS = Montgomery–Åsberg Depression Rating Scale; CGI = Clinical Global Impression scale; hsCRP = high-sensitivity C-reactive protein; IFN-γ = interferon gamma; IL = interleukin; TNF-α = tumor necrosis factor alpha; CMI = cell-mediated immunity; DTH = delayed-type hypersensitivity; NK = natural killer; PBMCs = peripheral blood mononuclear cells; COMT = catechol-O-methyltransferase; NPY = neuropeptide Y; E = epinephrine; MHPG = 3-methoxy-4-hydroxyphenylglycol; NE = norepinephrine; HVA = homovanillic acid; ELISA = enzyme-linked immunosorbent assay; GC–MS = gas chromatography–mass spectrometry; HPLC = high-performance liquid chromatography; RIA = radioimmunoassay.

**Table 2:** Characteristics of included studies investigating heart rate variability and immune markers in MDD.

First author (year)	Group	Medication status	Gender (%F)	Age M (SD)	BMI M(SD)	MDD scoring	Immune outcomes	ANS outcomes	HRV-immune associations	Method HRV
Carney et al. (2007)	44 MDD	/	40.90%	59.3 (9.8)	29.7 (5.9)	HDRS	IL-6, CRP, TNF-α, fibrinogen	LnTP, lnVLF, LnLF, LnHF	Fibrinogen: ↓ TP, VLF, LF, HF IL-6: ↓ TP, VLF, LF; – HF CRP and TNF-α: – TP, VLF, LF, HF	ECG during the night from PSG
Chang et al. (2017)	58 MDD + SI 61 MDD - SI	100% drug-naive	SI + group = 39.70% SI- group = 39.30%	SI + group = 32.41 (11.15), SI- group = 31.90 (12.77)	SI + group = 23.17 (4.22), SI- group = 21.94 (3.12)	HDRS-17	hsCRP, ESR	Mean RR intervals, variance (total HRV), VLF, LF, HF, LF/HF ratio	CRP (SI+): ↓ Var (SD of RR int), LF, HF; – mean RR interval, VLF, LF/HF ESR (SI+): – mean RR interval, Var (SD of RR int), VLF, LF, HF, LF/HF CRP (all): ↓ mean RR interval, HF; – Var (SD of RR int), VLF, LF, LF/HF ESR (all): ↓ LF; – mean RR interval, Var (SD of RR int), VLF, HF, LF/HF	5-min ECG
Tucker et al. (2018)	14 MDD-HS 4 MDD-C 20 HC-HS 30 HC-C	100% drug-naive	/	/	/	/	IL-6, IL-2	Total power (ms^2^), HF n.u., HF%, HF ms^2^, LF, LF/HF	IL-2: ↓ HF n.u., LF/HF; ↑ HF ms^2^; – Total power (ms^2^), HF%, LF ms^2^IL-6: – Total power (ms^2^), HF%, HF n.u., HF ms^2^, LF ms^2^, LF/HF	ECG (2 min baseline, 2 min test, 2 min recovery)
Hage et al. (2019)	64 MDD	100% drug-naive	65.63%	41.7 (11.9)	31.1 (6.4)	HAMD-17	IL-6, IL-8, IL-10, TNF-α, IL-1β, MCP-1, CRP	RSA, LF-HRV, HP	IL-6, IL-8, TNF-α, CRP, and MCP-1: – RSA, LF-HRV, HP IL-6: ↑ HP. IL-10: ↑ LF-HRV: – RSA, HP IL-1β: ↑ RSA; – LF-HRV, HP	15-min ECG
Buchmann et al. (2022)	70 MDE 195 past-MDE	100% drug -naive (6 = AD past 90 days)	60.5% HC 68.6% MDE	26.4 (5.6) MDE 24.1 (4.0) past-MDE	22.7 (3.8) MDE 22.7 (2.6) HC	BDI, MADRS, HAMD	TNF-α, IL-6	RSA CRP	RSA: ↓ TNF-α IL-6, IL-1a, IFNg CRP: – all HRV indices	PPG during 1-h MRI
Jangpangi et al. (2022)	30 MDD	100% drug-naive	53.30%	30.33 (6.97)	22.74 (1.75)	HDRS-24	IL-6, CRP	LF, HF, SDNN, RMSSD	CRP & IL-6: – LF, HF, SDNN, RMSSD	5-min ECG
Euteneuer et al. (2023)	80 MDD 40 HC	100% drug-naive	60% MDD 60% HC	30.31 (10.09) MDD 30.23 (9.63) HC	24.81 (4.50) MDD 22.72 (2.03) HC	BDI-II, MADRS	CRP, TNF-α, IL-6	HRV triangular index, SDNN, RMSSD, LF, HF (24h, daytime and nighttime)	CRP: – all HRV indices IL-6: ↓ Triangular index (24h, daytime), HF daytime, LF (24h, daytime), RMSSD daytime, SDNN 24h; – all other indices. TNF-α: – all HRV indices	24-h ECG
Singh et al. (2024)	65 MDD	100% drug-naive	41.70%	34.46 (7.13)	22.93 (2.30)	/	CRP	RMSSD, SDNN, pRR50, VLF, LF, HF, LF/HF, lnVLF, lnLF, lnHF	– RMSSD, SDNN, pRR50; ↑ VLF, LF, LF/HF, lnVLF, lnLF, lnLF/HF; ↓ HF, lnHF	5-min HRV ECG
Alimu et al. (2025)	20 MDD - LSD 34 MDD – MSD 23 MDD – SSD	100% drug-naive	68.06%	/	/	HAMD-24	WBC count, NEUT, LYMPH, CRP, ESR, IL-1β, IL-2R IL-6, IL-8, IL-10, TNF-α	LF, HF, LF/HF, SDNN, SDANN, SDNN index, RMSSD, pNN50, TRIIDX, Min HR, Max HR, Avg HR	WBC (SSD): ↑ MaxHR NEUT (SSD): ↑ LF, LF/HF; ↓ SDNN index, pNN50, TRIIDX CRP (SSD): ↑ LF/HF; ↓ HF IL-1β (SSD): ↑ SDANN, SDNN index IL-8 (SSD): ↓ LF. IL-6 (SSD): ↓ SDANN, TRI All other associations in SSD were non-significant; no significant correlations in LSD/MSD	24-h ECG

**Note.** ↓ = significant negative association; ↑ = significant positive association; – = no significant association. MDD = major depressive disorder; MDE = major depressive episode; SI = suicidal ideation; SSD = severe sleep disturbance; MSD = moderate sleep disturbance; LSD = low sleep disturbance; HC = healthy controls; AD = antidepressant; BDI = Beck Depression Inventory; BDI-II = Beck Depression Inventory II; MADRS = Montgomery–Åsberg Depression Rating Scale; HAM-D/HAMD-17/HDRS/HDRS-17/HDRS-21/HDRS-24 = Hamilton Depression Rating Scale (17, 21- or 24-item versions); BMI = body mass index; CRP = C-reactive protein; hsCRP = high-sensitivity C-reactive protein; ESR = erythrocyte sedimentation rate; IL = interleukin; IL-1β = interleukin-1 beta; IL-2 = interleukin-2; IL-2R = interleukin-2 receptor; IL-6 = interleukin-6; IL-8 = interleukin-8; IL-10 = interleukin-10; TNF-α = tumor necrosis factor alpha; MCP-1 = monocyte chemoattractant protein-1; WBC = white blood cell count; NEUT = neutrophil count; LYMPH = lymphocyte count; HRV = heart rate variability; RR interval = interval between successive R waves of the electrocardiogram; RMSSD = root mean square of successive differences; SDNN = standard deviation of NN intervals; SDANN = standard deviation of the averages of NN intervals; SDNN index = mean of the standard deviations of NN intervals calculated over short segments; pNN50/pRR50 = percentage of successive RR intervals differing by more than 50 ms; VLF = very-low-frequency power; LF = low-frequency power; HF = high-frequency power; LF/HF = ratio of low-to high-frequency power; LnTP = natural logarithm of total power; LnVLF = natural logarithm of very-low-frequency power; LnLF = natural logarithm of low-frequency power; LnHF = natural logarithm of high-frequency power; TRIIDX = HRV triangular index; RSA = respiratory sinus arrhythmia; LF-HRV = low-frequency heart rate variability; HP = heart period; Min HR = minimum heart rate; Max HR = maximum heart rate; Avg HR = average heart rate; ECG = electrocardiography; PPG = photoplethysmography; MRI = magnetic resonance imaging; PSG = polysomnography.

**Table 3:** Characteristics of included studies investigating vagus nerve function and immune markers in MDD.

First author (year)	Group	Medication status	MDD scoring	Gender (%F)	Age M (SD)	BMI M (SD)	Immune outcomes	ANS outcomes	Intervention
Pomara et al. (2021)	LLMD 27 HC 17	/	HDRS	33.3% MDD 58.8% HC	67.65 (5.36) LLMD 66.67 (5.36) HC	/	Serum IL-6, IL-8 CSF sTREM2, C3	CSF AChE, BChE	/
Kavakbasi et al. (2024)	TRD 6	/	MADRS	83%	47.8	/	hsCRP; IL-1β, −6, −8, −10, −12p, −17A, −18, −23, −33; IFN-α2, -γ; TNF-α; MCP-1	/	Cervical VNS treatment for 6 months
Lesperance et al. (2024)	TRD 6 HC 6	/	MADRS	67% TRD 67 HC	48.83 (41-57) MDD 32.67 (24-51) HC	/	IL-7, -6, -1β; TNF-α; IFN-γ; CXCL8; CCL2, -13, −17, −22; Flt-1; VEGF-C; bFGF	/	Cervical VNS treatment for 4 years
Scheller et al. (2025)	MDD 50 HC 50	100% AD	BDI	58% MDD 58 HC	45 (17) MDD 46 (21) HC	26.6 (5.7) MDD 24.5 (4.6) HC	IL-1 beta, IL-6, TNF-α, hsCRP	Average VN echogenicity (GSM-VN/GSM-CCA); HR, SDNN, RMSSD (5-min ECG)	/
Schiweck et al. (2025)	MDD 59 51 HC	(34/95) AD	MADRS BDI-II	63% MDD 65% HC	38.79 (13.29) MDD 30.24 (10.78) HC	24.32 (3.82) MDD 23.57 (2.59) HC	IL-6, TNF-α	RMSSD (5 min ECG)	taVNS or sham stimulation (∼90 min during experimental session)

**Note.** LLMD = late-life major depression; MDD = major depressive disorder; TRD = treatment-resistant depression; HAM-D/HDRS = Hamilton Depression Rating Scale; BDI = Beck Depression Inventory; BDI-II = Beck Depression Inventory-II; MADRS = Montgomery–Åsberg Depression Rating Scale; BMI = body mass index; IL = interleukin; TNF-α = tumor necrosis factor alpha; IFN-α2 = interferon alpha 2; IFN-γ = interferon gamma; MCP-1 = monocyte chemoattractant protein-1; CXCL = C-X-C motif chemokine ligand; CCL = C-C motif chemokine; Flt-1 = Fms-like tyrosine kinase-1; VEGF-C = vascular endothelial growth factor C; bFGF = basic fibroblast growth factor; hsCRP = high-sensitivity C-reactive protein; sTREM2 = soluble triggering receptor expressed on myeloid cells 2; C3 = complement component 3; CSF = cerebrospinal fluid; ANS = autonomic nervous system; HRV = heart rate variability; RMSSD = root mean square of successive differences; VN = vagus nerve; CCA = common carotid artery; GSM = grayscale mean; VNS = vagus nerve stimulation; taVNS = transcutaneous auricular vagus nerve stimulation; mMIST = Montreal Imaging Stress Task; LP = lumbar puncture; AChE = acetylcholinesterase; BChE = butyrylcholinesterase.

Substantial heterogeneity was observed in how ANS functioning was conceptualized and assessed. Accordingly, studies were grouped based on the autonomic branch and level of assessment, resulting in three overarching methodological domains. These included: (1) studies examining catecholaminergic signaling as molecular indicators of sympathetic nervous system (SNS) activity; (2) studies using cardiac physiological measures, such as HR and HRV as indices of overall autonomic regulation; and (3) studies focusing on parasympathetic (vagus nerve) functioning, assessed through indirect cholinergic markers and experimental modulation via vagus nerve stimulation (VNS). Detailed study characteristics for each domain are presented in Table 1, Table 2, Table 3.

### 3.2 Catecholamines

We identified 11 studies that investigated the catecholamines epinephrine (E) and norepinephrine (NE) in relation to the immune system in MDD. E and NE are the primary catecholamines of the SNS, playing a central role in mediating sympathetic activity. Sympathetic activation regulates catecholamine synthesis and releases catecholamines both at sympathetic nerve terminals and via the adrenal medulla, which is activated through preganglionic sympathetic input. While NE is predominantly released from sympathetic nerve endings, E is primarily secreted into the bloodstream by the adrenal medulla in response to sympathetic activation caused by stressful conditions (Esler et al., 1990; Won and Kim, 2016). Table 1 provides an overview of how SNS activity was measured across studies, including the biomarkers, biological matrices, measurement techniques, and the specific immune markers or functions assessed.

Two studies examined cross-sectional associations between circulating catecholamines or their metabolites and peripheral inflammatory markers. One study reported significant positive associations of interleukin (IL)-6 and CRP with NE, but independent of disease status, while another study found no associations between IL-6 and catecholamine metabolites (Yoshimura et al., 2019; Futtrup et al., 2018).

Studies utilizing acute stress paradigms found that stress-induced increases in E were positively associated with elevations in CRP and tumor necrosis factor alpha (TNF-α) in patients, but not in healthy controls (Weinstein et al., 2010). However, these associations were only examined at the time points when individual hormonal and inflammatory markers were statistically significant, rather than at standardized time points across markers. In contrast, another study found that stress-related changes in natural killer cell counts occurred independently of NE (Ravindran et al., 1996).

Several studies examined cell-mediated immune function in relation to catecholamines. Four studies found that higher catecholaminergic activity was associated with altered lymphocyte function, including lower lymphocyte proliferative responses (Maes et al., 1989) and reduced delayed-type hypersensitivity skin responses (Hickie et al., 1995). In the third study, E induced dose-dependent suppression of T cell proliferation in both MDD and healthy controls; however, individuals with MDD exhibited reduced sensitivity to E, reflected in a higher proportion of nonresponders compared to controls (Lopes et al., 2012). One study examining noradrenaline transporter density and intracellular catecholamine metabolism in lymphocytes found evidence of a decreased catecholaminergic turnover rate compared to controls, but these cellular measures were not related to depression severity (Mata et al., 2005).

In contrast to these inhibitory findings, one study reported that plasma NE concentrations were positively associated with mitogen-stimulated IL-2 production in individuals with MDD and dysthymia, but not in healthy controls (Anisman et al., 1999). Two studies reported no associations between catecholaminergic activity and cell-mediated immune functions (Irwin et al., 1991, 2003).

### 3.3 Cardiac physiological measurements

HR and HRV are widely used, non-invasive markers of ANS function, reflecting the combined influence of sympathetic and parasympathetic activity on cardiac regulation. To facilitate interpretation, an overview of the HR(V) indices assessed across the included studies is provided in Supplementary Table S1.

Across the included cross-sectional studies, results were heterogeneous not only in terms of inflammatory markers and clinical subgroups but also with respect to the HR(V) indices used and regarding the direction and strength of reported associations. Many studies assessed multiple HR(V) indices spanning parasympathetic-related, mixed autonomic, and global measures, alongside a range of immune markers, including both pro-inflammatory and regulatory/anti-inflammatory markers, which complicates direct comparison and synthesis of findings. An overview of the specific HR(V) indices measured, HR(V) recording methods, recording durations, analyzed immune markers, and reported HR(V)–immune associations is provided in Table 2, including both significant and non-significant findings. To maintain clarity and readability, only statistically significant associations are discussed in the main text.

Four studies found negative correlations between inflammatory markers and vagally mediated HRV indices such as the root mean square of successive differences (RMSSD), high-frequency power (HF), and respiratory sinus arrhythmia (RSA); however, it is important to note that these studies did not adjust for covariates (Singh et al., 2024; Buchmann et al., 2022; Chang et al., 2017; Carney et al., 2007). In studies adjusting for covariates, evidence was more limited. One study reported that both daytime HF and RMSSD were significantly associated with IL-6 levels (Euteneuer et al., 2023), while another found significant associations between the percentage of successive normal-to-normal intervals differing by more than 50 ms (pNN50) and both white blood cell count and IL-1β (Alimu et al., 2025). In addition, one study examined HR and found that IL-6 was positively associated with minimum HR after covariate adjustment (Buchmann et al., 2022).

Regarding mixed measures of autonomic activity, one study reported positive correlations between CRP and frequency-domain indices, including very low-frequency power (VLF), and the low-frequency to high-frequency ratio (LF/HF), although these analyses were unadjusted. In contrast, three studies reported inverse associations, including inverse associations between CRP and mean RR interval, erythrocyte sedimentation rate and low-frequency power (LF) (Chang et al., 2017), as well as between LF, VLF, and total power (TP) with IL-6 and fibrinogen, without adjustment (Carney et al., 2007), and one study reporting a negative association between LF and IL-8 after adjustment (Alimu et al., 2025).

Positive associations were also reported in two studies. One study found unadjusted associations between heart period and IL-6 (Hage et al., 2019), and another study between the standard deviation of normal-to-normal intervals (SDNN) and IL-8, SDNN and CRP, IL-1β and HF, and IL-6 and the standard deviation of the average normal-to-normal intervals (SDANN), after adjustment (Alimu et al., 2025).

Two studies also examined cytokines with mixed or anti-inflammatory properties. IL-2 was positively associated with HF and negatively associated with LF/HF (Tucker et al., 2018), while IL-10 was positively associated with LF, both in unadjusted analyses (Hage et al., 2019).

One study took circadian variation of HRV into account by examining associations separately for 24-h, daytime, and nighttime measures. In this study, IL-6 was inversely correlated with the HRV triangular index (24-h and daytime), as well as with LF (24-h and daytime), RMSSD (daytime), HF (daytime), and SDNN (24-h), after covariate adjustment (Euteneuer et al., 2023).

Stratified analyses further indicated that HRV–immune associations were more pronounced in specific clinical subgroups, including patients characterized by differing levels of sleep disturbance (Alimu et al., 2025). These associations differed across sleep subgroups in a marker-specific manner, with the strongest effects observed in the severe sleep disturbance group. Similar subgroup-specific effects were observed in MDD with suicidal ideation, where stronger negative associations between CRP and HF and LF were reported in comparison with MDD without suicidal ideation (Chang et al., 2017).

Only one study compared individuals with current MDD with those in remission and reported stronger associations between RSA and cytokines in the current MDD group, with larger effect sizes observed for IL-1α, IL-6, and TNF-α; notably, the association with IL-1α reached significance only in the current MDD group (Buchmann et al., 2022). However, mediation analyses from the same study indicated that cytokines did not significantly mediate the relationship between RSA and depressive symptoms. Another study found that depression severity did not strengthen the correlation between IL-6 and HRV indices (Jangpangi et al., 2022).

### 3.4 Vagus nerve functioning

The parasympathetic nervous system, primarily mediated by the vagus nerve, is an important regulator of immune activity through cholinergic signaling. Acetylcholine (ACh), the parasympathetic neurotransmitter, modulates inflammatory responses via the CAIP, in which vagal efferent signaling and ACh-dependent activation of α7 nicotinic ACh receptors (α7nAChR) on monocytes and macrophages result in reduced cytokine release (Pavlov et al., 2003). CAIP has been investigated using indirect markers of cholinergic function, as ACh itself is highly labile and rapidly degraded, making it difficult to quantify directly. Consequently, studies use enzymes involved in its breakdown, such as acetylcholinesterase (AChE) and butyrylcholinesterase (BChE), as indirect markers of cholinergic activity (Pavlov et al., 2009; Zygmunt and Stanczyk, 2010). In addition, VNS, which delivers chronic electrical stimulation to the vagus nerve, has been used as an experimental approach to assess its anti-inflammatory effects (Borovikova et al., 2000; Huston and Tracey, 2011). Table 3 provides an overview of how parasympathetic (vagal) activity was assessed across studies, including the ANS measures, immune markers, and VNS methodologies.

One study examined cholinergic regulation in relation to inflammatory effects in late-life MDD and reported that CSF AChE and BChE levels were negatively associated with IL-6, while both MDD and healthy controls showed positive associations with central markers of microglial activation and neuroinflammation (Pomara et al., 2021).

Three studies examined the effects of long-term VNS on immune parameters in patients with treatment-resistant or difficult-to-treat depression. One study followed six patients over 12 months and reported non-significant trends toward differential cytokine trajectories between responders and nonresponders, with lower cytokine levels at six months in patients who responded clinically at 12 months (Kavakbasi et al., 2024). Another study reported significant modulation of multiple inflammatory proteins after more than four years of continuous VNS, including reductions in cytokines and chemokines involved in leukocyte recruitment and changes in markers related to blood–brain barrier regulation (Lesperance et al., 2024). In contrast, a study of transcutaneous auricular VNS (taVNS) during a stress paradigm found no overall effects on TNF-α and IL-6 when comparing individuals with MDD and healthy controls directly; however, stratification based on baseline RMSSD revealed slight TNF-α reductions in individuals with low RMSSD and opposite inflammatory effects in individuals with high RMSSD (Schiweck et al., 2025).

One study examined associations between inflammatory cytokines, vagus nerve morphology, and resting HRV in patients with MDD. Although initial analyses showed significant correlations between hsCRP and HR or HRV indices in the MDD group, these associations did not remain significant after adjustment for BMI (Scheller et al., 2025).

## 4 Discussion

### 4.1 ANS–immune interactions in MDD

#### 4.1.1 Sympathetic activity and immune function in MDD

Findings on catecholamine–immune interactions were inconsistent across studies, with some evidence suggesting altered associations in individuals with MDD compared to healthy controls.

The heterogeneous associations between catecholamines and immune markers observed across studies in depression likely reflect (Liu et al., 2020a) the context-dependent nature of SNS activation and (Monroe and Anderson, 2015) substantial methodological heterogeneity in how catecholaminergic activity is measured and interpreted. Experimental studies on cell-mediated immunity similarly reported both inhibitory and stimulatory effects of catecholamines, consistent with the context-dependent nature of adrenergic signaling. The net effect of adrenergic receptor stimulation on immune cells is highly context-dependent, varying according to factors such as receptor subtype expression, local catecholamine concentration, and proximity to sympathetic nerve terminals (Elenkov et al., 2000; Pongratz and Straub, 2014; Grassi and Esler, 1999). Consequently, sympathetic signaling can exert both pro- and anti-inflammatory effects. For example, high local concentrations of NE preferentially activate β-adrenergic receptors, resulting in anti-inflammatory effects, whereas lower concentrations favor α-adrenergic signaling and pro-inflammatory responses (Pongratz and Straub, 2014).

Methodological heterogeneity further contributes to inconsistent findings. Studies differ in biological matrices, analytes, analytical techniques, and sampling protocols, and given the short half-life and context sensitivity of catecholamines, these approaches likely capture different aspects of sympathetic activity (Grassi and Esler, 1999; McCance, 1991; Oberbeck, 2006; Goldstein et al., 2003). For example, plasma catecholamine concentrations reflect sympathetic activity at the time of sampling and can capture rapid responses to acute stress, whereas urinary catecholamines change more slowly and provide an integrated measure of sympathoadrenal activity over the collection period. Moreover, plasma concentrations may be influenced by the sampling site and regional catecholamine release and clearance, while urinary concentrations are additionally affected by renal function (Steptoe, 1987). This methodological heterogeneity is further illustrated by studies included in this review using acute stress paradigms, where one study examined associations at marker-specific peak responses, whereas another evaluated pre– and post–changes across the stress paradigm.

Within-study comparisons provided preliminary evidence that catecholamine–immune coupling may be altered in MDD in comparison with healthy controls. Persistent or repeated sympathoadrenal activation could cause β_2_-adrenergic receptor downregulation, receptor internalization, or impaired receptor downstream signaling (Sanders and Kohm, 2002). This may explain why one study found that individuals with MDD showed reduced sensitivity to the suppressive effect of E on T-cell proliferation, with a higher proportion of nonresponders than among healthy controls. Evidence from panic disorder provides a potentially relevant parallel, with studies reporting reduced lymphocyte β-adrenergic receptor density and impaired β-receptor responsiveness (Brown et al., 1988; Maddock et al., 1993). These results make receptor desensitization plausible across stress-related disorders such as MDD. Altered noradrenaline-transporter density and reduced intracellular catecholamine turnover in lymphocytes provide additional evidence of disrupted cellular catecholamine regulation in MDD. However, these measures reflect catecholamine handling rather than adrenergic receptor responsiveness and were not associated with depression severity.

#### 4.1.2 Cardiac autonomic markers and systemic inflammation

Several HRV-immune studies reported that higher levels of pro-inflammatory markers were associated with lower vagally mediated HRV indices, including RMSSD, HF, pNN50, and RSA. In line with this pattern, IL-6 was also positively associated with HR, reflecting reduced vagal or relatively increased sympathetic influence. These findings align with meta-analytic evidence across broader populations, demonstrating robust inverse associations between vagally mediated HRV and inflammatory markers, supporting the existence of a general relationship between reduced vagal regulation and increased inflammatory markers rather than one specific to MDD (Williams et al., 2019). Nevertheless, these associations may be more pronounced in depression, particularly given that depression severity has also been associated with both higher inflammatory marker levels (Foley et al., 2021) and reduced HRV (Kemp et al., 2010).

Importantly, none of the included studies directly compared the strength of HRV–immune associations between individuals with MDD and healthy controls; therefore, it remains uncertain whether these associations are more pronounced in MDD. One study reported stronger associations between cytokines and RSA in individuals with current MDD compared to those in remission. Another study did not find evidence that depression severity strengthened the associations between IL-6 and HRV indices, although it used categorical severity groups and included only a small subgroup with severe depression (n = 6), which may have limited its statistical power to detect stronger inflammation–HRV associations. In a study with patients with coronary heart disease, inverse associations between HRV and CRP were stronger among those with elevated depressive symptoms, whereas differences for IL-6 were not significant (Frasure-Smith et al., 2009). This may partly reflect that CRP is a more stable marker of systemic inflammation, while circulating IL-6 is more transient. This CRP-HRV finding suggests that ANS–immune alterations may be dimensionally related to depressive symptom burden across clinical populations rather than being specific to MDD.

Important to mention is that several studies did not adjust for covariates known to influence both HRV and immune markers, such as age, sex, BMI, physical activity, smoking, hypertension, medication use, and comorbid diseases (Aeschbacher et al., 2017; Lampert et al., 2008). This suggests that reported associations may be confounded and should be interpreted with caution, as they may not reflect independent relationships between HRV and immune markers.

Heterogeneity in HRV findings may also arise due to its high sensitivity to methodological differences, including variation in measurement modality (e.g., ECG vs. PPG) (Schafer and Vagedes, 2013), sampling rate (Burma et al., 2021), body posture (Fei et al., 1994), and recording duration (Billman, 2013). Regarding the latter, studies used both short-term and long-term recordings that capture different aspects of autonomic regulation. Only one study accounted for circadian variation in HRV, which may better capture dynamic ANS dysfunction in MDD than traditional mean HRV metrics (Li et al., 2024).

#### 4.1.3 Parasympathetic regulation and vagal modulation of inflammation

One study found that lower CSF AChE and BChE levels were associated with increased IL-6. This inverse relationship between cholinesterase levels and peripheral inflammatory markers is broadly consistent with findings in other populations, suggesting that CAIP activity may be upregulated as a compensatory response to systemic inflammation (Markuskova et al., 2023). Similarly, increased pro-inflammatory markers have been associated with upregulation of α7nAChR at both protein and mRNA levels, further supporting this compensatory hypothesis (Diaz-Marsa et al., 2012; MacDowell et al., 2013; Xu et al., 2019; Zivkovic et al., 2015).

In contrast, within the same study, higher levels of CSF AChE and BChE were positively associated with CSF markers of microglial activation and neuroinflammation. ACh exerts anti-inflammatory and neuroprotective effects in the central nervous system (CNS) by modulating glial cells, particularly microglia, via α7nAChR signaling. A hypo-cholinergic state, potentially resulting from increased cholinesterase activity, may reduce ACh availability and thereby contribute to increased neuroinflammatory signaling (Li et al., 2019). The opposing associations may reflect compartment-specific cholinergic regulation, with increased cholinesterase activity indicating reduced ACh-mediated anti-inflammatory control in the CNS, while in the periphery, it possibly reflects a compensatory upregulation of the CAIP in response to systemic inflammation.

However, neither cholinesterase levels nor α7nAChR expression directly measures CAIP activity, and increased receptor expression does not necessarily indicate increased downstream signaling. Mechanistically, preclinical evidence suggests that taVNS in MDD may activate auricular vagal afferents projecting through the nucleus tractus solitarius to the hypothalamus and amygdala (Liu et al., 2020b), where α7nAChR-dependent JAK2/STAT3 signaling may suppress NF-κB-mediated microglial activation and pro-inflammatory cytokine production (Wang et al., 2025), but this mechanism is derived primarily from animal studies and has not been demonstrated directly in patients. The compensatory and compartment-specific interpretations therefore remain hypothetical.

This translational gap is further complicated because CAIP, including in the context of VNS, is typically studied in controlled preclinical settings using young, healthy animals exposed to acute immune challenges, with VNS often administered prophylactically or shortly after inflammation induction. In contrast, clinical studies typically involve older patients with chronic, treatment-resistant conditions, in which cytokines are commonly used as proxy markers of CAIP activation despite being non-specific and not providing direct evidence of pathway engagement.

The three VNS studies differed substantially in methodology, which may partly explain the heterogeneous findings. Two studies used chronically implanted cervical VNS, whereas the third used acute non-invasive taVNS, likely engaging partly distinct vagal pathways (Kaniusas et al., 2019). One cervical VNS study applied individualized stimulation settings tailored to each participant, another did not report the stimulation parameters in detail, and the taVNS study used a standardized stimulation protocol and a sham condition. In addition, the studies differed considerably in the chronicity and timing of stimulation and immune assessment, ranging from acute single-session paradigms to long-term stimulation over months or years. A meta-analysis examining the effects of VNS on inflammatory markers across different clinical populations also found inconsistent results, indicating that responses may vary between diseases because of differences in pathophysiology and within diagnostic groups because of variation in disease subtype (Schiweck et al., 2024).

Moreover, VNS is often implicitly equated with activation of the CAIP, which may oversimplify its mechanisms of action and obscure the potential involvement of non-CAIP neuroimmune pathways. Accordingly, one proposed model suggests that taVNS may improve MDD not only through the CAIP but also by modulating the brain–gut and hypothalamic–pituitary–adrenal (HPA) axis, the default mode network, the salience network, and reward circuitry. Differential activation of these pathways may therefore contribute to the heterogeneous inflammatory outcomes observed in clinical studies (Liu et al., 2020b; Schiweck et al., 2024; Reardon, 2024).

### 4.2 Future implications

#### 4.2.1 Methodological considerations

Taken together, these findings highlight several methodological considerations for future research. Early research already indicated that no single measurement adequately captures SNS activity, and combining multiple techniques or measurements is recommended to understand SNS function better (Grassi and Esler, 1999; McCance, 1991). Because SNS markers often reflect transient sympathetic activation rather than stable autonomic regulation, repeated or longitudinal measurements are recommended instead of relying on a single assessment. Because the effects of the SNS on immune function are broad and context-dependent, studies should carefully select SNS and immune markers that best reflect the specific SNS–immune interaction they want to investigate.

Future studies should also consider how broader physiological systems may shape ANS–immune interactions. Although the HPA axis and gut microbiome were outside the predefined scope of this review, both are closely interconnected with autonomic and immune regulation. The HPA axis interacts with both the sympathetic–adrenal–medullary system, which mediates the rapid sympathetic stress response through the release of E and NE (LeMoult, 2020), and the immune system. One pathway underlying its interaction with the immune system involves cortisol, the end product of the HPA axis, which acts on glucocorticoid receptors expressed by immune cells. Reduced receptor expression or sensitivity in MDD may weaken cortisol's anti-inflammatory effects, thereby contributing to increased inflammation (Amasi-Hartoonian et al., 2022). The gut microbiome may provide an additional pathway: preclinical evidence indicates that dysbiosis can alter inflammatory activity and gut vagal innervation, potentially through immune-cell recruitment or activation (Kim et al., 2020). Simultaneously assessing HPA-axis, autonomic, microbiome, and immune markers may therefore help clarify how disturbances in neuroendocrine–immune communication contribute to the pathophysiology of MDD.

HRV represents one of the most widely applied functional measures of autonomic regulation. However, substantial heterogeneity exists in how HRV is measured, analyzed, and reported. To improve methodological rigor and transparency, guidelines have been developed that provide recommendations for standardized HRV data acquisition, preprocessing, analysis, and reporting (Quintana et al., 2016; Laborde et al., 2017). Complementing these recommendations, recent work has highlighted that many HRV indices are highly correlated and capture overlapping physiological processes; therefore, selecting a single representative index per HRV domain may reduce redundancy and improve comparability between studies (Pham et al., 2025). Given that different HRV indices reflect distinct aspects of autonomic regulation, future work should examine how specific HRV domains relate to different immune processes rather than treating HRV as a unitary construct. In addition, using circadian HRV analyses may improve the assessment of dynamic ANS–immune interactions by capturing diurnal variation rather than relying solely on mean HRV values.

Notably, almost none of the studies included in this review examined HR itself, despite evidence suggesting that HR demonstrates high classification accuracy for MDD and that elevated HR has been associated with increased inflammatory markers (Schiweck et al., 2021; Whelton et al., 2014). Future research should therefore include HR alongside HRV to obtain a more comprehensive assessment of autonomic functioning in MDD.

Regarding the CAIP, the available clinical evidence remains insufficient to establish whether observed immune changes reflect activation of this pathway specifically in MDD. Limited evidence suggests that central and peripheral cholinergic activity should be distinguished, as cholinesterase levels and other biomarkers may reflect different processes and components of the pathway across compartments. Building on this, future intervention studies targeting the CAIP, such as VNS, should adopt mechanistically informed and standardized sham-controlled designs to assess CAIP-specific pathway engagement and distinguish it from other VNS-related mechanisms. In addition, longitudinal assessment of immune markers and systematic testing of how different VNS settings influence outcomes, rather than applying a single fixed stimulation protocol to all patients, are needed to better understand the relationship between VNS-induced immune changes and treatment response.

As many of the studies included in this review were observational and cross-sectional and often involved small sample sizes, larger longitudinal studies should be conducted to examine the temporal relationship between the immune system and the ANS. In addition, future studies should more systematically compare healthy controls and individuals with MDD with regard to ANS–immune associations, while also accounting for differences in depression severity, as this may help clarify whether and how these relationships vary across levels of symptom burden and are specifically altered in the context of MDD.

#### 4.2.2 Implications for stratification

The heterogeneity observed across studies suggests that ANS–immune dysregulation may not be uniform across all individuals with MDD, but may instead characterize biologically distinct subgroups. In this review, stronger cytokine–HRV associations were observed in current versus remitted MDD, while associations also differed across sleep disturbance and suicidal ideation subgroups. Broader literature similarly indicates that autonomic and inflammatory alterations vary across depressive subtypes. For example, melancholic depression is more strongly associated with reduced HRV and elevated HR (Kemp et al., 2014), and atypical depression is moreassociated with higher inflammatory markers (Lamers et al., 2013). These findings suggest that ANS–immune alterations may relate more closely to specific biological and symptom profiles than to the diagnostic category itself, highlighting the importance of biologically informed stratification approaches in future research.

In the context of interventions such as VNS, mechanistically informed stratification may also be important. Two of the VNS studies included in this review did not stratify participants based on baseline inflammatory or autonomic status, which may have obscured potential subgroup-specific effects and reduced the ability to detect meaningful immunological changes. One study included in this review investigating taVNS in MDD found no overall differences in RMSSD and inflammation when comparing individuals with MDD and healthy controls. However, when participants were stratified across diagnostic groups based on baseline RMSSD, individuals with low RMSSD showed improved cardiac activity and reduced inflammation, whereas opposite effects were observed in individuals with high RMSSD. These findings suggest that baseline autonomic functioning may represent a more informative biological stratification marker for taVNS response than diagnostic status alone (Schiweck et al., 2025).

Sex may represent an additional source of heterogeneity, as previous research suggests that the association between HRV and inflammatory markers may differ according to sex, with some evidence of stronger associations in women than in men (Williams et al., 2019). Conversely, a larger study found no significant sex differences in HRV–inflammation associations after covariate adjustment (Cooper et al., 2015). Although some included studies in this review adjusted for sex, only one reported sex-stratified associations, but did not formally test whether ANS–immune relationships differed by sex. Therefore, no conclusions regarding sex-specific differences can be drawn. Future studies should use adequately powered sex-stratified analyses and formal interaction tests to determine whether ANS–immune relationships vary according to sex.

### 4.3 Limitations

This scoping review has several limitations. First, no formal quality appraisal or risk-of-bias assessment was conducted; therefore, the findings should be interpreted as a mapping of the available evidence rather than conclusions about the strength or validity of individual associations. Second, the evidence base was relatively small, fragmented, and highly heterogeneous in terms of study design, population characteristics, ANS measures, and immune markers. Third, the review focused primarily on ANS–immune interactions and did not systematically integrate other stress-related biological systems, such as the HPA axis and gut microbiota, which also interact with autonomic and immune pathways.

### 4.4 Conclusion

This scoping review mapped the current evidence on the relationship between ANS functioning and immune processes in MDD. Overall, findings provide some indication that alterations in ANS functioning may be associated with immune dysregulation in individuals with MDD compared with healthy controls; however, results across studies were highly heterogeneous.

This heterogeneity likely reflects both biological complexity and methodological variation across studies. In addition, limited comparisons of ANS–immune associations with healthy controls and consideration of depression severity restrict understanding of whether these findings reflect MDD-specific dysregulation. Future studies should prioritize larger controlled longitudinal designs to examine temporal and potentially causal relationships between ANS functioning and the immune system in MDD, alongside greater transparency and standardization in measurement approaches and the use of mechanistically informed intervention designs, such as stratified VNS studies.

Taken together, this review provides a structured overview of a complex and evolving field and underscores the need for more research. A more focused systematic review may be warranted as the evidence grows and becomes more methodologically consistent.

## CRediT authorship contribution statement

**Emma Saillart:** Conceptualization, Investigation, Methodology, Visualization, Writing – original draft, Writing – review & editing. **Katleen Jacobs:** Investigation, Methodology, Writing – review & editing. **Stephan Claes:** Conceptualization, Writing – review & editing. **Elfi Vergaelen:** Conceptualization, Investigation, Writing – review & editing.

## Declaration of generative AI and AI-assisted technologies in the manuscript preparation process

During the final stages of manuscript preparation, the authors used ChatGPT in order to improve the readability and language of the text. After using this tool/service, the author(s) reviewed and edited the content as needed and take(s) full responsibility for the content of the published article.

## Source of funding

Emma Saillart is supported by a TBM (Toegepast Biomedisch Onderzoek) grant from the Research Foundation – Flanders (FWO) [grant number T001222N] awarded to Stephan Claes (KU Leuven).

## Declaration of competing interests

The authors declare that they have no known competing financial interests or personal relationships that could have appeared to influence the work reported in this paper.

## Data Availability

No data was used for the research described in the article.
