# Supplementary material for: The interaction between the immune system and the autonomic nervous system in the pathophysiology of major depressive disorder: a scoping review
Source: Brain Behav Immun Health. 2026 Aug 22;56:101334. doi: 10.1016/j.bbih.2026.101334 (PMC13527485; doi:10.1016/j.bbih.2026.101334)
Supplement: Supplementary file 1 [file mmc1.docx]

**Supplementary file 1**

The search strategy combined three core concepts across all databases: (1) major depressive disorder, (2) autonomic nervous system, and (3) immune system. Controlled vocabulary terms and free-text keywords were adapted to the syntax and indexing system of each database. The full search strings for all databases are provided in this supplementary file.

Combination of concepts for all databases:

Concept 1 AND Concept 2 AND Concept 3

## **Pubmed (including Medline, via NCBI)**

**Concept 1:**

"Depressive Disorder"[Mesh] OR “MDD”[tiab] OR “depression”[tiab] OR “depressive disorder*”[tiab]

**Concept 2:**

"Sympathetic Nervous System"[Mesh:NoExp] OR "Parasympathetic Nervous System"[Mesh:NoExp] OR "Heart Rate"[Mesh] OR "Vagus Nerve"[Mesh:NoExp] OR “Acetylcholine”[Mesh] OR ”Catecholamines”[Mesh:NoExp] OR "Blood Pressure"[Mesh:NoExp] OR “Arterial Pressure”[Mesh:NoExp] OR “Sympathoadrenal System”[Mesh:NoExp] OR "autonomous"[tiab] OR “autonomic”[tiab] OR "sympathetic"[tiab] OR "parasympathetic"[tiab] OR ANS[tiab] OR PNS[tiab] OR SNS[tiab] OR "heartbeat*"[tiab] OR "heart beat*"[tiab] OR "heart rate*"[tiab] OR HRV[tiab] OR “R-R interval”[tiab] OR “RR interval”[tiab] OR “catecholamine*”[tiab] OR ((“Epinephrine"[Mesh:NoExp] OR "Norepinephrine"[Mesh:NoExp] OR "epinephrine"[tiab] OR "norepinephrine"[tiab] OR “adrenalin”[tiab] OR “noradrenalin”[tiab]) AND ("blood"[Subheading] OR "urine"[Subheading] OR “metabolism”[Subheading] OR "blood*"[tiab] OR “serum”[tiab] OR “plasma”[tiab] OR "urin*"[tiab] OR "stress*"[tiab] OR “metabolite*”[tiab] OR “biomarker*”[tiab] OR “bio-marker*”[tiab] OR “peripheral”[tiab] OR “hormone*”[tiab])) OR "acetylcholine"[tiab] OR "sympathetic–adrenal–medullary axis"[tiab] OR SAM[tiab] OR "RR variability"[tiab] OR "stress hormone*"[tiab] OR "vagus"[tiab] OR "vagal"[tiab] OR "blood pressure"[tiab] OR “arterial pressure”[tiab] OR "fight-or-flight"[tiab] OR "respiratory sinus arrhythmia"[tiab] OR "RSA"[tiab] OR “sympathoadrenal”[tiab] OR “sympatho adrenal”[tiab] OR “cholinergic”[tiab] OR “cranial nerve”[tiab] OR “pulse pressure”[tiab] OR “polyvagal”[tiab] OR “pre-ejection period”[tiab]

**Concept 3:**

**“**Immune System”[Mesh] OR “Inflammation”[Mesh:NoExp] OR "Cytokines"[Mesh] OR “Immunoproteins”[Mesh] OR “Leukocytes”[Mesh] OR "Lipopolysaccharides"[Mesh:NoExp] OR “Microglia”[Mesh:NoExp] OR “Neopterin”[Mesh:NoExp] OR “Fibrinogen”[Mesh:NoExp] OR “E-selectin”[Mesh:NoExp] OR "Serum Amyloid A Protein"[Mesh:NoExp] OR "Blood Sedimentation"[Mesh:NoExp] OR "Platelet Count"[Mesh] OR "Albumins"[Mesh:NoExp] OR "Serum Albumin"[Mesh:NoExp] OR "HMGB1 Protein"[Mesh:NoExp] OR "NF-kappa B"[Mesh:NoExp] OR "Vascular Endothelial Growth Factors"[Mesh:NoExp] OR “E-selectin”[tiab] OR "immun*"[tiab] OR “neuroimmun*”[tiab] OR “neuroinflamma*”[tiab] OR "inflamma*"[tiab] OR "interleukin*”[tiab] OR “cytokine*”[tiab] OR “chemokine*”[tiab] OR “CCL”[tiab] OR “CXCL”[tiab] OR “CX3C*”[tiab] OR “C-reactive protein”[tiab] OR “CRP”[tiab] OR “lipopolysaccharide*”[tiab] OR “LPS”[tiab] OR “IL-1*”[tiab] OR “IL-2*”[tiab] OR “IL-3*”[tiab] OR “IL-4*”[tiab] OR “IL-5”[tiab] OR “IL-6”[tiab] OR “IL-7”[tiab] OR “IL-8”[tiab] OR “IL-9”[tiab] OR "IL1"[tiab] OR "IL2"[tiab] OR "IL3"[tiab] OR "IL4"[tiab] OR "IL5"[tiab] OR "IL6"[tiab] OR "IL7"[tiab] OR "IL8"[tiab] OR "IL9"[tiab] OR "IL10"[tiab] OR "IL11"[tiab] OR "IL12"[tiab] OR "IL13"[tiab] OR "IL14"[tiab] OR "IL15"[tiab] OR "IL16"[tiab] OR "IL17"[tiab] OR "IL18"[tiab] OR "IL19"[tiab] OR "IL20"[tiab] OR "IL21"[tiab] OR "IL22"[tiab] OR "IL23"[tiab] OR "IL24"[tiab] OR "IL25"[tiab] OR "IL26"[tiab] OR "IL27"[tiab] OR "IL28"[tiab] OR "IL29"[tiab] OR "IL30"[tiab] OR "IL31"[tiab] OR "IL32"[tiab] OR "IL33"[tiab] OR "IL34"[tiab] OR "IL35"[tiab] OR "IL36"[tiab] OR "IL37"[tiab] OR "IL38"[tiab] OR "IL39"[tiab] OR "IL40"[tiab] OR “IgG”[tiab] OR “IgM”[tiab] OR “IgA”[tiab] OR “IgE”[tiab] OR “IgD”[tiab] OR “lymphocyte*”[tiab] OR “white blood cell*” [tiab] OR “WBC” [tiab] OR “leukocyte*”[tiab] OR “B-cell*”[tiab] OR “T-cell*”[tiab] OR “T-helper”[tiab] OR “th17“[tiab] OR “th-17“[tiab] OR “th1”[tiab] OR “th2”[tiab] OR “th-1”[tiab] OR “th-2”[tiab] OR “neutrophil*”[tiab] OR “CD4”[tiab] OR “CD8”[tiab] OR “basophil*”[tiab] OR “eosinophil*”[tiab] OR “granulocyte*”[tiab] OR “peripheral mononuclear blood cell*”[tiab] OR “PBMC*”[tiab] OR “monocyte*”[tiab] OR “lymphocyte*”[tiab] OR “interferon*”[tiab] OR “IFN”[tiab] OR “IFNγ”[tiab] OR “IFNα”[tiab] OR “IFNβ”[tiab] OR “IFNg”[tiab] OR “IFNa”[tiab] OR “IFNb”[tiab] OR “tumor necrosis factor*”[tiab] OR “TNF”[tiab] OR “TNFα”[tiab] OR “TNFa”[tiab] OR “sTNF*”[tiab] OR “transforming growth factor*”[tiab] OR “TGF”[tiab] OR “TGFα”[tiab] OR “TGFβ”[tiab] OR “TGFa”[tiab] OR “TGFb”[tiab] OR “macrophage*”[tiab] OR “natural killer cell*”[tiab] OR “NK-cell*”[tiab] OR “dendritic cell*”[tiab] OR “mast cell*”[tiab] OR “plasma cell*”[tiab] OR “antigen-presenting cell*”[tiab] OR “APC”[tiab] OR “lymphoid*”[tiab] OR “microglia*”[tiab] OR "neopterin"[tiab] OR "fibrinogen"[tiab] OR "E-selectin"[tiab] OR "serum amyloid A"[tiab] OR "SAA"[tiab] OR "erythrocyte sedimentation rate"[tiab] OR "ESR"[tiab] OR “platelet count”[tiab] OR “albumin”[tiab] OR “high mobility group box 1”[tiab] OR “HMGB1”[tiab] OR “HMGB-1”[tiab] OR “nuclear factor-kappa B”[tiab] OR “NF-κB”[tiab] OR “NF-kB”[tiab] OR “NFκB”[tiab] OR “NFkB”[tiab] OR “NF-kappa*”[tiab] OR “NFkappa*”[tiab] OR  “vascular endothelial growth factor”[tiab] OR “VEGF”[tiab]

## **EMBASE (via embase.com)**

**Concept 1:**

'depression'/exp OR ‘MDD’:ti,ab,kw OR ‘depression*’:ti,ab,kw OR ‘depressive disorder*’:ti,ab,kw

**Concept 2:**

'adrenergic system'/de OR 'cholinergic system'/de OR 'heart rate and rhythm'/exp OR 'vagus nerve'/de OR ‘acetylcholine’/de OR ‘catecholamine’/de OR ‘blood pressure’/de OR 'systolic blood pressure'/de OR 'diastolic blood pressure'/de OR ‘arterial pressure’/de OR ‘pulse pressure’/de OR ‘fight or flight response’/de OR 'pre-ejection period'/de OR ‘autonomous’:ti,ab,kw OR ‘autonomic’:ti,ab,kw OR 'sympathetic':ti,ab,kw OR 'parasympathetic':ti,ab,kw OR ANS:ti,ab,kw OR PNS:ti,ab,kw OR SNS:ti,ab,kw OR 'heartbeat*':ti,ab,kw OR 'heart beat*':ti,ab,kw OR 'heart rate*':ti,ab,kw OR HRV:ti,ab,kw OR ‘R-R interval’:ti,ab,kw OR ‘RR interval’:ti,ab,kw OR ‘catecholamine*’:ti,ab,kw OR ((‘epinephrine’/de OR ‘noradrenalin’/de OR 'epinephrine':ti,ab,kw OR 'norepinephrine':ti,ab,kw OR ‘adrenalin’:ti,ab,kw OR ‘noradrenalin’:ti,ab,kw) AND ('blood*':ti,ab,kw OR 'serum':ti,ab,kw OR 'plasma':ti,ab,kw OR 'urin*':ti,ab,kw OR 'stress*':ti,ab,kw OR 'metabolite*':ti,ab,kw OR 'biomarker*':ti,ab,kw OR ‘bio-marker*’:ti,ab,kw OR 'peripheral':ti,ab,kw OR ‘hormone*’:ti,ab,kw)) OR 'acetylcholine':ti,ab,kw OR 'sympathetic–adrenal–medullary axis':ti,ab,kw OR SAM:ti,ab,kw OR 'RR variability':ti,ab,kw OR 'stress hormone*':ti,ab,kw OR 'vagus':ti,ab,kw OR 'vagal':ti,ab,kw OR 'blood pressure':ti,ab,kw OR ‘arterial pressure’:ti,ab,kw OR 'fight-or-flight':ti,ab,kw OR 'respiratory sinus arrhythmia':ti,ab,kw OR 'RSA':ti,ab,kw OR 'sympathoadrenal':ti,ab,kw OR 'sympatho-adrenal':ti,ab,kw OR 'cholinergic':ti,ab,kw OR 'cranial nerve':ti,ab,kw OR 'pulse pressure':ti,ab,kw OR ‘polyvagal’:ti,ab,kw OR ‘pre-ejection period’:ti,ab,kw

**Concept 3:**

'Immune System'/exp OR ‘inflammation’/de OR 'nervous system inflammation'/de OR ‘cytokine’/exp OR ‘immunoglobulin’/exp OR ‘leukocyte’/exp OR 'lipopolysaccharide'/de OR 'neopterin'/de OR 'fibrinogen'/de OR 'endothelial leukocyte adhesion molecule 1'/de OR 'serum amyloid A'/de OR 'erythrocyte sedimentation rate'/de OR 'platelet count'/de OR 'albumin'/de OR 'high mobility group B1 protein'/de OR 'immunoglobulin enhancer binding protein'/de OR 'vasculotropin'/de OR 'immun*':ti,ab,kw OR ‘neuroimmun*’:ti,ab,kw OR ’neuroinflamma*’:ti,ab,kw OR 'inflamma*':ti,ab,kw OR 'interleukin*':ti,ab,kw OR 'cytokine*':ti,ab,kw OR 'chemokine*':ti,ab,kw OR 'C-reactive protein':ti,ab,kw OR 'CRP':ti,ab,kw OR 'lipopolysaccharide*':ti,ab,kw OR 'LPS':ti,ab,kw OR 'IL-1*':ti,ab,kw OR 'IL-2*':ti,ab,kw OR 'IL-3*':ti,ab,kw OR 'IL-4*':ti,ab,kw OR 'IL-5':ti,ab,kw OR 'IL-6':ti,ab,kw OR 'IL-7':ti,ab,kw OR 'IL-8':ti,ab,kw OR 'IL-9':ti,ab,kw OR 'IL1':ti,ab,kw OR 'IL2':ti,ab,kw OR 'IL3':ti,ab,kw OR 'IL4':ti,ab,kw OR 'IL5':ti,ab,kw OR 'IL6':ti,ab,kw OR 'IL7':ti,ab,kw OR 'IL8':ti,ab,kw OR 'IL9':ti,ab,kw OR 'IL10':ti,ab,kw OR 'IL11':ti,ab,kw OR 'IL12':ti,ab,kw OR 'IL13':ti,ab,kw OR 'IL14':ti,ab,kw OR 'IL15':ti,ab,kw OR 'IL16':ti,ab,kw OR 'IL17':ti,ab,kw OR 'IL18':ti,ab,kw OR 'IL19':ti,ab,kw OR 'IL20':ti,ab,kw OR 'IL21':ti,ab,kw OR 'IL22':ti,ab,kw OR 'IL23':ti,ab,kw OR 'IL24':ti,ab,kw OR 'IL25':ti,ab,kw OR 'IL26':ti,ab,kw OR 'IL27':ti,ab,kw OR 'IL28':ti,ab,kw OR 'IL29':ti,ab,kw OR 'IL30':ti,ab,kw OR 'IL31':ti,ab,kw OR 'IL32':ti,ab,kw OR 'IL33':ti,ab,kw OR 'IL34':ti,ab,kw OR 'IL35':ti,ab,kw OR 'IL36':ti,ab,kw OR 'IL37':ti,ab,kw OR 'IL38':ti,ab,kw OR 'IL39':ti,ab,kw OR 'IL40':ti,ab,kw OR 'IgG':ti,ab,kw OR 'IgM':ti,ab,kw OR 'IgA':ti,ab,kw OR 'IgE':ti,ab,kw OR 'IgD':ti,ab,kw OR 'lymphocyte*':ti,ab,kw OR 'white blood cell*':ti,ab,kw OR ‘WBC*':ti,ab,kw OR 'leukocyte*':ti,ab,kw OR 'B-cell*':ti,ab,kw OR 'T-cell*':ti,ab,kw OR ‘T-helper’:ti,ab,kw OR ‘th17’:ti,ab,kw OR ‘th-17’:ti,ab,kw OR ‘th1’:ti,ab,kw OR ‘th-1’:ti,ab,kw OR ‘th2’:ti,ab,kw OR ‘th-2’:ti,ab,kw OR 'neutrophil*':ti,ab,kw OR 'CD4':ti,ab,kw OR 'CD8':ti,ab,kw OR 'basophil*':ti,ab,kw OR 'eosinophil*':ti,ab,kw OR 'granulocyte*':ti,ab,kw OR 'peripheral mononuclear blood cell*':ti,ab,kw OR 'PBMC*':ti,ab,kw OR 'monocyte*':ti,ab,kw OR 'interferon*':ti,ab,kw OR 'IFN*':ti,ab,kw OR 'tumor necrosis factor*':ti,ab,kw OR 'TNF*’:ti,ab,kw OR 'sTNF*':ti,ab,kw OR ‘transforming growth factor*’:ti,ab,kw OR ‘TGF*’:ti,ab,kw OR 'macrophage*':ti,ab,kw OR 'natural killer cell*':ti,ab,kw OR 'NK-cell*':ti,ab,kw OR 'dendritic cell*':ti,ab,kw OR 'mast cell*':ti,ab,kw OR 'plasma cell*':ti,ab,kw OR 'antigen-presenting cell*':ti,ab,kw OR 'APC':ti,ab,kw OR 'lymphoid*':ti,ab,kw OR 'microglia*':ti,ab,kw OR 'neopterin':ti,ab,kw OR 'fibrinogen':ti,ab,kw OR 'E-selectin':ti,ab,kw OR 'serum amyloid A':ti,ab,kw OR 'SAA':ti,ab,kw OR 'erythrocyte sedimentation rate':ti,ab,kw OR 'ESR':ti,ab,kw OR 'platelet count':ti,ab,kw OR 'albumin':ti,ab,kw OR 'high mobility group box 1':ti,ab,kw OR 'HMGB1':ti,ab,kw OR 'HMGB-1':ti,ab,kw OR 'nuclear factor-kappa B':ti,ab,kw OR 'NF-κB':ti,ab,kw OR 'NF-kB':ti,ab,kw OR ‘NF kappa*’:ti,ab,kw OR 'NFκB':ti,ab,kw OR 'NFkB':ti,ab,kw OR ‘NFkappa*’:ti,ab,kw OR 'vascular endothelial growth factor':ti,ab,kw OR 'VEGF':ti,ab,kw

## **Web of Science core collection (webofscience.com)**

- Science Citation Index Expanded (SCI-EXPANDED) -- 1955-present
- Social Sciences Citation Index (SSCI) -- 1956-present
- Arts & Humanities Citation Index (AHCI) -- 1975-present
- Conference Proceedings Citation Index – Science (CPCI-S) -- 1990-present
- Conference Proceedings Citation Index – Social Science & Humanities (CPCI-SSH) -- 1990-present
- Emerging Sources Citation Index (ESCI) -- 2019-present

**Concept 1:**

TS=("MDD" OR "depression*" OR "depressive disorder*")

**Concept 2:**

TS=("autonomous" OR “autonomic” OR "sympathetic" OR "parasympathetic" OR “ANS” OR “PNS” OR “SNS” OR "heartbeat*" OR "heart beat*" OR "heart rate*" OR “HRV” OR “R-R interval” OR “RR interval” OR “catecholamine*” OR (("epinephrine" OR "norepinephrine" OR “adrenalin” OR “noradrenalin”) AND ("blood*" OR “serum” OR “plasma” OR "urin*" OR "stress*" OR “metabolite*” OR “biomarker*” OR “bio marker*” OR “peripheral” OR “hormone*”)) OR "acetylcholine" OR "sympathetic–adrenal–medullary axis" OR “SAM” OR "RR variability" OR "stress hormone*" OR "vagus" OR "vagal" OR "blood pressure" OR “arterial pressure” OR "fight-or-flight" OR "respiratory sinus arrhythmia" OR "RSA" OR “sympathoadrenal” OR “sympatho adrenal” OR “cholinergic” OR “cranial nerve” OR “pulse pressure” OR “polyvagal” OR “pre-ejection period”)

**Concept 3:**

TS=("immun*" OR “neuroimmun*” OR “neuroinflamma*” OR "inflamma*" OR "interleukin*" OR “cytokine*” OR “chemokine*” OR “CCL” OR “CXCL” OR “CX3C*” OR “C-reactive protein” OR “CRP” OR “lipopolysaccharide*” OR “LPS” OR “IL-1*” OR “IL-2*” OR “IL-3*” OR “IL-4*” OR “IL-5” OR “IL-6” OR “IL-7” OR “IL-8” OR “IL-9” OR "IL1" OR "IL2" OR "IL3" OR "IL4" OR "IL5" OR "IL6" OR "IL7" OR "IL8" OR "IL9" OR "IL10" OR "IL11" OR "IL12" OR "IL13" OR "IL14" OR "IL15" OR "IL16" OR "IL17" OR "IL18" OR "IL19" OR "IL20" OR "IL21" OR "IL22" OR "IL23" OR "IL24" OR "IL25" OR "IL26" OR "IL27" OR "IL28" OR "IL29" OR "IL30" OR "IL31" OR "IL32" OR "IL33" OR "IL34" OR "IL35" OR "IL36" OR "IL37" OR "IL38" OR "IL39" OR "IL40" OR “IgG” OR “IgM” OR “IgA” OR “IgE” OR “IgD” OR “lymphocyte*” OR “white blood cell*” OR “WBC” OR “leukocyte*” OR “B-cell*” OR “T-cell*” OR “T-helper” OR “th17“ OR “th-17“ OR “th1” OR “th-1” OR “th2” OR “th-2” OR “neutrophil*” OR “CD4” OR “CD8” OR “basophil*” OR “eosinophil*” OR “granulocyte*” OR “peripheral mononuclear blood cell*” OR “PBMC*” OR “monocyte*” OR “lymphocyte*” OR “interferon*” OR “IFN*” OR “tumor necrosis factor*” OR “TNF*” OR “sTNF*” OR “transforming growth factor*” OR “TGF*” OR “macrophage*” OR “natural killer cell*” OR “NK-cell*” OR “dendritic cell*” OR “mast cell*” OR “plasma cell*” OR “antigen-presenting cell*” OR “APC” OR “lymphoid*” OR “microglia*” OR "neopterin" OR "fibrinogen" OR "E-selectin" OR "serum amyloid A" OR "SAA" OR "erythrocyte sedimentation rate" OR "ESR" OR “platelet count” OR “albumin” OR “high mobility group box 1” OR “HMGB1” OR “HMGB-1” OR “nuclear factor-kappa B” OR “NF-κB” OR “NF-kB” OR “NF kappa*” OR “NFκB” OR “NFkB” OR “NFkappa*” OR “vascular endothelial growth factor” OR “VEGF”)

## **Scopus (via scopus.com)**

**Concept 1:**

(TITLE-ABS ("MDD" OR "depression*" OR "depressive disorder*")

OR

AUTHKEY ("MDD" OR "depression*" OR "depressive disorder*"))

**Concept 2:**

(TITLE-ABS ("autonomous" OR “autonomic” OR "sympathetic" OR "parasympathetic" OR ANS OR PNS OR SNS OR "heartbeat*" OR "heart beat*" OR "heart rate*" OR HRV OR “R-R interval” OR “RR interval” OR “catecholamine*” OR (("epinephrine" OR "norepinephrine" OR “adrenalin” OR “noradrenalin”) AND ("blood*" OR “serum” OR “plasma” OR "urin*" OR "stress*" OR “metabolite*” OR “biomarker*” OR “bio-marker*” OR “peripheral” OR “hormone*”)) OR "acetylcholine" OR "sympathetic–adrenal–medullary axis" OR SAM OR "RR variability" OR "stress hormone*" OR "vagus" OR "vagal" OR "blood pressure" OR “arterial pressure” OR "fight-or-flight" OR "respiratory sinus arrhythmia" OR "RSA" OR “sympathoadrenal” OR “sympatho adrenal” OR “cholinergic” OR “cranial nerve” OR “pulse pressure” OR “polyvagal” OR “pre-ejection period”)

OR

AUTHKEY ("autonomous" OR “autonomic” OR "sympathetic" OR "parasympathetic" OR ANS OR PNS OR SNS OR "heartbeat*" OR "heart beat*" OR "heart rate*" OR HRV OR “R-R interval” OR “RR interval” OR “catecholamine*” OR (("epinephrine" OR "norepinephrine" OR “adrenalin” OR “noradrenalin”) AND ("blood*" OR “serum” OR “plasma” OR "urin*" OR "stress*" OR “metabolite*” OR “biomarker*” OR “bio-marker*” OR “peripheral” OR “hormone*”)) OR "acetylcholine" OR "sympathetic–adrenal–medullary axis" OR SAM OR "RR variability" OR "stress hormone*" OR "vagus" OR "vagal" OR "blood pressure" OR “arterial pressure” OR "fight-or-flight" OR "respiratory sinus arrhythmia" OR "RSA" OR “sympathoadrenal” OR “sympatho adrenal” OR “cholinergic” OR “cranial nerve” OR “pulse pressure” OR “polyvagal” OR “pre-ejection period”))

**Concept 3:**

(TITLE-ABS ("immun*" OR “neuroimmun*” OR “neuroinflamma*” OR "inflamma*" OR "interleukin*" OR “cytokine*” OR “chemokine*” OR “CCL” OR “CXCL” OR “CX3C*” OR “C-reactive protein” OR “CRP” OR “lipopolysaccharide*” OR “LPS” OR “IL-1*” OR “IL-2*” OR “IL-3*” OR “IL-4*” OR “IL-5” OR “IL-6” OR “IL-7” OR “IL-8” OR “IL-9” OR "IL1" OR "IL2" OR "IL3" OR "IL4" OR "IL5" OR "IL6" OR "IL7" OR "IL8" OR "IL9" OR "IL10" OR "IL11" OR "IL12" OR "IL13" OR "IL14" OR "IL15" OR "IL16" OR "IL17" OR "IL18" OR "IL19" OR "IL20" OR "IL21" OR "IL22" OR "IL23" OR "IL24" OR "IL25" OR "IL26" OR "IL27" OR "IL28" OR "IL29" OR "IL30" OR "IL31" OR "IL32" OR "IL33" OR "IL34" OR "IL35" OR "IL36" OR "IL37" OR "IL38" OR "IL39" OR "IL40" OR “IgG” OR “IgM” OR “IgA” OR “IgE” OR “IgD” OR “lymphocyte*” OR “white blood cell*” OR “WBC” OR “leukocyte*” OR “B-cell*” OR “T-cell*” OR “T-helper” OR “th17“ OR “th-17“ OR “th1” OR “th-1” OR “th2” OR “th-2” OR “neutrophil*” OR “CD4” OR “CD8” OR “basophil*” OR “eosinophil*” OR “granulocyte*” OR “peripheral mononuclear blood cell*” OR “PBMC*” OR “monocyte*” OR “lymphocyte*” OR “interferon*” OR “IFN*” OR “tumor necrosis factor*” OR “TNF*” OR “sTNF*” OR “transforming growth factor*” OR “TGF*” OR “macrophage*” OR “natural killer cell*” OR “NK-cell*” OR “dendritic cell*” OR “mast cell*” OR “plasma cell*” OR “antigen-presenting cell*” OR “APC” OR “lymphoid*” OR “microglia*” OR "neopterin" OR "fibrinogen" OR "E-selectin" OR "serum amyloid A" OR "SAA" OR "erythrocyte sedimentation rate" OR "ESR" OR “platelet count” OR “albumin” OR “high mobility group box 1” OR “HMGB1” OR “HMGB-1” OR “nuclear factor-kappa B” OR “NF-*B” OR “NF*b” OR “NF kappa*” OR “NFkappa*” OR “vascular endothelial growth factor” OR “VEGF”)

OR

AUTHKEY ("immun*" OR “neuroimmun*” OR “neuroinflamma*” OR "inflamma*" OR "interleukin*" OR “cytokine*” OR “chemokine*” OR “CCL” OR “CXCL” OR “CX3C*” OR “C-reactive protein” OR “CRP” OR “lipopolysaccharide*” OR “LPS” OR “IL-1*” OR “IL-2*” OR “IL-3*” OR “IL-4*” OR “IL-5” OR “IL-6” OR “IL-7” OR “IL-8” OR “IL-9” OR "IL1" OR "IL2" OR "IL3" OR "IL4" OR "IL5" OR "IL6" OR "IL7" OR "IL8" OR "IL9" OR "IL10" OR "IL11" OR "IL12" OR "IL13" OR "IL14" OR "IL15" OR "IL16" OR "IL17" OR "IL18" OR "IL19" OR "IL20" OR "IL21" OR "IL22" OR "IL23" OR "IL24" OR "IL25" OR "IL26" OR "IL27" OR "IL28" OR "IL29" OR "IL30" OR "IL31" OR "IL32" OR "IL33" OR "IL34" OR "IL35" OR "IL36" OR "IL37" OR "IL38" OR "IL39" OR "IL40" OR “IgG” OR “IgM” OR “IgA” OR “IgE” OR “IgD” OR “lymphocyte*” OR “white blood cell*” OR “WBC” OR “leukocyte*” OR “B-cell*” OR “T-cell*” OR “T-helper” OR “th17“ OR “th-17“ OR “th1” OR “th-1” OR “th2” OR “th-2” OR “neutrophil*” OR “CD4” OR “CD8” OR “basophil*” OR “eosinophil*” OR “granulocyte*” OR “peripheral mononuclear blood cell*” OR “PBMC*” OR “monocyte*” OR “lymphocyte*” OR “interferon*” OR “IFN*” OR “tumor necrosis factor*” OR “TNF*” OR “sTNF*” OR “transforming growth factor*” OR “TGF*” OR “macrophage*” OR “natural killer cell*” OR “NK-cell*” OR “dendritic cell*” OR “mast cell*” OR “plasma cell*” OR “antigen-presenting cell*” OR “APC” OR “lymphoid*” OR “microglia*” OR "neopterin" OR "fibrinogen" OR "E-selectin" OR "serum amyloid A" OR "SAA" OR "erythrocyte sedimentation rate" OR "ESR" OR “platelet count” OR “albumin” OR “high mobility group box 1” OR “HMGB1” OR “HMGB-1” OR “nuclear factor-kappa B” OR “NF-*B” OR “NF*B” OR “NF kappa*” OR “NFkappa*” OR “vascular endothelial growth factor” OR “VEGF”))

## **CENTRAL (via Cochrane Library)**

**#1:** [mh "Depressive Disorder"]

**#2:** ("MDD" OR "depression" OR (depressive NEXT disorder*)):ti,ab,kw

**#3:** #1 OR #2

**#4** [mh ^"Sympathetic Nervous System"] OR [mh ^"Parasympathetic Nervous System"] OR [mh "Heart Rate"] OR [mh ^"Vagus Nerve"] OR [mh “Acetylcholine”] OR [mh ^“Catecholamines”] OR [mh ^"Blood Pressure"] OR [mh ^“Arterial Pressure”] OR [mh ^“Sympathoadrenal System”]

**#5:** ("autonomous" OR “autonomic” OR "sympathetic" OR "parasympathetic" OR ANS OR PNS OR SNS OR heartbeat* OR (heart NEXT beat*) OR (heart NEXT rate*) OR HRV OR (R-R NEXT interval*) OR (RR NEXT interval*) OR catecholamine* OR (("epinephrine" OR "norepinephrine" OR “adrenalin” OR “noradrenalin”) AND (blood* OR “serum” OR “plasma” OR urin* OR stress* OR metabolite* OR biomarker* OR (bio NEXT marker*) OR “peripheral” OR hormone*)) OR "acetylcholine" OR "sympathetic–adrenal–medullary axis" OR SAM OR "RR variability" OR (stress NEXT hormone*) OR "vagus" OR "vagal" OR "blood pressure" OR “arterial pressure” OR "fight-or-flight" OR "respiratory sinus arrhythmia" OR "RSA" OR “sympathoadrenal” OR “sympatho adrenal” OR “cholinergic” OR “cranial nerve” OR “pulse pressure” OR “polyvagal” OR “pre-ejection period”):ti,ab,kw OR (([mh ^"Epinephrine"] OR [mh ^"Norepinephrine"]) AND (blood* OR “serum” OR “plasma” OR urin* OR stress* OR metabolite* OR biomarker* OR (bio NEXT marker*) OR “peripheral” OR hormone*):ti,ab,kw)

**#6: #4 OR #5**

**#7** [mh "Immune System"] OR [mh ^"Inflammation"] OR [mh "Cytokines"] OR [mh "Immunoproteins"] OR [mh "Leukocytes"] OR [mh ^"Lipopolysaccharides"] OR [mh ^"Microglia"] OR [mh ^"Neopterin"] OR [mh ^"Fibrinogen"] OR [mh ^"E-selectin"] OR [mh ^"Serum Amyloid A Protein"] OR [mh ^"Blood Sedimentation"] OR [mh "Platelet Count"] OR [mh ^"Albumins"] OR [mh ^"Serum Albumin"] OR [mh ^"HMGB1 Protein"] OR [mh ^"NF-kappa B"] OR [mh ^"Vascular Endothelial Growth Factors"]

**#8** (immun* OR neuroimmun* OR neuroinflamma* OR inflamma* OR interleukin* OR cytokine* OR chemokine* OR “CCL” OR “CXCL” OR CX3C* OR “C-reactive protein” OR “CRP” OR lipopolysaccharide* OR “LPS” OR IL-1* OR IL-2* OR IL-3* OR IL-4* OR “IL-5” OR “IL-6” OR “IL-7” OR “IL-8” OR “IL-9” OR "IL1" OR "IL2" OR "IL3" OR "IL4" OR "IL5" OR "IL6" OR "IL7" OR "IL8" OR "IL9" OR "IL10" OR "IL11" OR "IL12" OR "IL13" OR "IL14" OR "IL15" OR "IL16" OR "IL17" OR "IL18" OR "IL19" OR "IL20" OR "IL21" OR "IL22" OR "IL23" OR "IL24" OR "IL25" OR "IL26" OR "IL27" OR "IL28" OR "IL29" OR "IL30" OR "IL31" OR "IL32" OR "IL33" OR "IL34" OR "IL35" OR "IL36" OR "IL37" OR "IL38" OR "IL39" OR "IL40" OR “IgG” OR “IgM” OR “IgA” OR “IgE” OR “IgD” OR lymphocyte* OR (“white blood” NEXT cell*) OR “WBC” OR leukocyte* OR B-cell* OR T-cell* OR “T-helper” OR “th17” OR (th NEXT “17”) OR “th1” OR (th NEXT “1”) OR “th2” OR (th NEXT “2”) OR neutrophil* OR “CD4” OR “CD8” OR basophil* OR eosinophil* OR granulocyte* OR (“peripheral mononuclear blood” NEXT cell*) OR PBMC* OR monocyte* OR lymphocyte* OR interferon* OR IFN* OR (“tumor necrosis” NEXT factor*) OR TNF* OR sTNF* OR (“transforming growth” NEXT factor*) OR TGF* OR macrophage* OR (“natural killer” NEXT cell*) OR NK-cell* OR (dendritic NEXT cell*) OR (mast NEXT cell*) OR (plasma NEXT cell*) OR (“antigen-presenting” NEXT cell*) OR “APC” OR lymphoid* OR microglia* OR "neopterin" OR "fibrinogen" OR "E-selectin" OR "serum amyloid A" OR "SAA" OR "erythrocyte sedimentation rate" OR "ESR" OR “platelet count” OR “albumin” OR “high mobility group box 1” OR “HMGB1” OR “HMGB 1” OR “nuclear factor-kappa B” OR (NF NEXT *B) OR NF*B OR (NF NEXT kappa*) OR “vascular endothelial growth factor” OR “VEGF”):ti,ab,kw

## **CINAHL (via EBSCOhost)**

**Concept 1 :**

(MH "Depression+") OR AB ("MDD" OR "depression" OR "depressive disorder*") OR TI ("MDD" OR "depression" OR "depressive disorder*")

**Concept 2 :**

(MH "Sympathetic Nervous System") OR (MH "Parasympathetic Nervous System") OR (MH "Heart Rate") OR (MH "Vagus Nerve") OR (MH "Heart Rate Variability") OR (MH "Acetylcholine") OR (MH "Catecholamines") OR (MH "Blood Pressure") OR (MH "Systolic Pressure") OR (MH "Diastolic Pressure") OR (MH "Arterial Pressure") OR AB ("autonomous" OR “autonomic” OR "sympathetic" OR "parasympathetic" OR ANS OR PNS OR SNS OR "heartbeat*" OR "heart beat*" OR "heart rate*" OR HRV OR “R-R interval” OR “RR interval” OR “catecholamine*” OR "acetylcholine" OR "sympathetic–adrenal–medullary axis" OR SAM OR "RR variability" OR "stress hormone*" OR "vagus" OR "vagal" OR "blood pressure" OR “arterial pressure” OR "fight-or-flight" OR "respiratory sinus arrhythmia" OR "RSA" OR “sympathoadrenal” OR “sympatho adrenal” OR “cholinergic” OR “cranial nerve” OR “pulse pressure” OR “polyvagal” OR “pre-ejection period”) OR TI ("autonomous" OR “autonomic” OR "sympathetic" OR "parasympathetic" OR ANS OR PNS OR SNS OR "heartbeat*" OR "heart beat*" OR "heart rate*" OR HRV OR “R-R interval” OR “RR interval” OR “catecholamine*” OR "acetylcholine" OR "sympathetic–adrenal–medullary axis" OR SAM OR "RR variability" OR "stress hormone*" OR "vagus" OR "vagal" OR "blood pressure" OR “arterial pressure” OR "fight-or-flight" OR "respiratory sinus arrhythmia" OR "RSA" OR “sympathoadrenal” OR “sympatho adrenal” OR “cholinergic” OR “cranial nerve” OR “pulse pressure” OR “polyvagal” OR “pre-ejection period”) OR (((MH "Epinephrine+") OR TI ("epinephrine" OR "norepinephrine" OR “adrenalin” OR “noradrenalin”) OR AB ("epinephrine" OR "norepinephrine" OR “adrenalin” OR “noradrenalin”)) AND (TI ("blood*" OR “serum” OR “plasma” OR "urin*" OR "stress*" OR “metabolite*” OR “biomarker*” OR “bio marker*” OR “peripheral” OR “hormone*”) OR AB ("blood*" OR “serum” OR “plasma” OR "urin*" OR "stress*" OR “metabolite*” OR “biomarker*” OR “bio marker*” OR “peripheral” OR “hormone*”)))

**Concept 3:**

(MH "Immune System+") OR (MH "Inflammation") OR (MH "Neuroinflammation") OR (MH "Cytokines+") OR (MH "Immunoproteins+”) OR (MH "Leukocytes+") OR (MH "Lipopolysaccharides") OR (MH "Fibrinogen") OR (MH "Blood Sedimentation") OR (MH "Platelet Count") OR (MH "Albumins") OR (MH "Serum Albumin") OR (MH "NF-kappa B") OR (MH "Vascular Endothelial Growth Factors") OR AB ("immun*" OR “neuroimmun*” OR “neuroinflamma*” OR "inflamma*" OR "interleukin*" OR “cytokine*” OR “chemokine*” OR “CCL” OR “CXCL” OR “CX3C*” OR “C-reactive protein” OR “CRP” OR “lipopolysaccharide*” OR “LPS” OR “IL-1*” OR “IL-2*” OR “IL-3*” OR “IL-4*” OR “IL-5” OR “IL-6” OR “IL-7” OR “IL-8” OR “IL-9” OR "IL1" OR "IL2" OR "IL3" OR "IL4" OR "IL5" OR "IL6" OR "IL7" OR "IL8" OR "IL9" OR "IL10" OR "IL11" OR "IL12" OR "IL13" OR "IL14" OR "IL15" OR "IL16" OR "IL17" OR "IL18" OR "IL19" OR "IL20" OR "IL21" OR "IL22" OR "IL23" OR "IL24" OR "IL25" OR "IL26" OR "IL27" OR "IL28" OR "IL29" OR "IL30" OR "IL31" OR "IL32" OR "IL33" OR "IL34" OR "IL35" OR "IL36" OR "IL37" OR "IL38" OR "IL39" OR "IL40" OR “IgG” OR “IgM” OR “IgA” OR “IgE” OR “IgD” OR “lymphocyte*” OR “white blood cell*” OR “WBC” OR “leukocyte*” OR “B-cell*” OR “T-cell*” OR “T-helper” OR “th17“ OR “th 17“ OR “th1” OR “th 1” OR “th2” OR “th 2” OR “neutrophil*” OR “CD4” OR “CD8” OR “basophil*” OR “eosinophil*” OR “granulocyte*” OR “peripheral mononuclear blood cell*” OR “PBMC*” OR “monocyte*” OR “lymphocyte*” OR “interferon*” OR “IFN*” OR “tumor necrosis factor*” OR “TNF*” OR “sTNF*” OR “transforming growth factor*” OR “TGF*” OR “macrophage*” OR “natural killer cell*” OR “NK-cell*” OR “dendritic cell*” OR “mast cell*” OR “plasma cell*” OR “antigen-presenting cell*” OR “APC” OR “lymphoid*” OR “microglia*” OR "neopterin" OR "fibrinogen" OR "E-selectin" OR "serum amyloid A" OR "SAA" OR "erythrocyte sedimentation rate" OR "ESR" OR “platelet count” OR “albumin” OR “high mobility group box 1” OR “HMGB1” OR “HMGB 1” OR “nuclear factor-kappa B” OR “NF-*B” OR “NF*b” OR “NF kappa*” OR “NFkappa*” OR “vascular endothelial growth factor” OR “VEGF”) OR AB ("immun*" OR “neuroimmun*” OR “neuroinflamma*” OR "inflamma*" OR "interleukin*" OR “cytokine*” OR “chemokine*” OR “CCL” OR “CXCL” OR “CX3C*” OR “C-reactive protein” OR “CRP” OR “lipopolysaccharide*” OR “LPS” OR “IL-1*” OR “IL-2*” OR “IL-3*” OR “IL-4*” OR “IL-5” OR “IL-6” OR “IL-7” OR “IL-8” OR “IL-9” OR "IL1" OR "IL2" OR "IL3" OR "IL4" OR "IL5" OR "IL6" OR "IL7" OR "IL8" OR "IL9" OR "IL10" OR "IL11" OR "IL12" OR "IL13" OR "IL14" OR "IL15" OR "IL16" OR "IL17" OR "IL18" OR "IL19" OR "IL20" OR "IL21" OR "IL22" OR "IL23" OR "IL24" OR "IL25" OR "IL26" OR "IL27" OR "IL28" OR "IL29" OR "IL30" OR "IL31" OR "IL32" OR "IL33" OR "IL34" OR "IL35" OR "IL36" OR "IL37" OR "IL38" OR "IL39" OR "IL40" OR “IgG” OR “IgM” OR “IgA” OR “IgE” OR “IgD” OR “lymphocyte*” OR “white blood cell*” OR “WBC” OR “leukocyte*” OR “B-cell*” OR “T-cell*” OR “T-helper” OR “th17“ OR “th 17“ OR “th1” OR “th 1” OR “th2” OR “th 2” OR “neutrophil*” OR “CD4” OR “CD8” OR “basophil*” OR “eosinophil*” OR “granulocyte*” OR “peripheral mononuclear blood cell*” OR “PBMC*” OR “monocyte*” OR “lymphocyte*” OR “interferon*” OR “IFN*” OR “tumor necrosis factor*” OR “TNF*” OR “sTNF*” OR “transforming growth factor*” OR “TGF*” OR OR “macrophage*” OR “natural killer cell*” OR “NK-cell*” OR “dendritic cell*” OR “mast cell*” OR “plasma cell*” OR “antigen-presenting cell*” OR “APC” OR “lymphoid*” OR “microglia*” OR "neopterin" OR "fibrinogen" OR "E-selectin" OR "serum amyloid A" OR "SAA" OR "erythrocyte sedimentation rate" OR "ESR" OR “platelet count” OR “albumin” OR “high mobility group box 1” OR “HMGB1” OR “HMGB 1” OR “nuclear factor-kappa B” OR “NF-*B” OR “NF*b” OR “NF kappa*” OR “NFkappa*” OR “vascular endothelial growth factor” OR “VEGF”)

## **PsycArticles (via ProQuest)**

**Concept 1**

MAINSUBJECT.EXACT.EXPLODE("Persistent Depressive Disorder") OR MAINSUBJECT.EXACT.EXPLODE("Major Depression") OR MAINSUBJECT.EXACT("Affective Disorders") OR TI,AB,IF("MDD" OR "depression*" OR "depressive disorder*")

**Concept 2**

MAINSUBJECT.EXACT("Parasympathetic Nervous System") OR MAINSUBJECT.EXACT("Sympathetic Nervous System”) OR MAINSUBJECT.EXACT("Heart Rate") OR MAINSUBJECT.EXACT("Heart Rate Variability") OR MAINSUBJECT.EXACT("Vagus Nerve") OR MAINSUBJECT.EXACT("Epinephrine") OR MAINSUBJECT.EXACT("Norepinephrine") OR MAINSUBJECT.EXACT("Acetylcholine") OR MAINSUBJECT.EXACT("Catecholamines") OR MAINSUBJECT.EXACT.EXPLODE("Blood Pressure") OR TI,AB,IF("autonomous" OR “autonomic” OR "sympathetic" OR "parasympathetic" OR “ANS” OR “PNS” OR “SNS” OR "heartbeat*" OR "heart beat*" OR "heart rate*" OR “HRV” OR “R-R interval” OR “RR interval” OR “catecholamine*” OR (("epinephrine" OR "norepinephrine" OR “adrenalin” OR “noradrenalin”) AND ("blood*" OR “serum” OR “plasma” OR "urin*" OR "stress*" OR “metabolite*” OR “biomarker*” OR “bio marker*” OR “peripheral” OR “hormone*”)) OR "acetylcholine" OR "sympathetic–adrenal–medullary axis" OR “SAM” OR "RR variability" OR "stress hormone*" OR "vagus" OR "vagal" OR "blood pressure" OR “arterial pressure” OR "fight-or-flight" OR "respiratory sinus arrhythmia" OR "RSA" OR “sympathoadrenal” OR “sympatho adrenal” OR “cholinergic” OR “cranial nerve” OR “pulse pressure” OR “polyvagal” OR “pre-ejection period”)

**Concept 3:**

MAINSUBJECT.EXACT("Immune System") OR MAINSUBJECT.EXACT("Inflammation") OR MAINSUBJECT.EXACT("Neuroinflammation") OR MAINSUBJECT.EXACT.EXPLODE("Cytokines") OR MAINSUBJECT.EXACT.EXPLODE("Immunoglobulins") OR MAINSUBJECT.EXACT.EXPLODE("Leucocytes") OR MAINSUBJECT.EXACT("C-Reactive Protein") OR MAINSUBJECT.EXACT("Lipopolysaccharide") OR MAINSUBJECT.EXACT("Microglia") OR MAINSUBJECT.EXACT("Serum Albumin") OR TI,AB,IF("immun*" OR “neuroimmun*” OR “neuroinflamma*” OR "inflamma*" OR "interleukin*" OR “cytokine*” OR “chemokine*” OR “CCL” OR “CXCL” OR “CX3C*” OR “C-reactive protein” OR “CRP” OR “lipopolysaccharide*” OR “LPS” OR “IL-1*” OR “IL-2*” OR “IL-3*” OR “IL-4*” OR “IL-5” OR “IL-6” OR “IL-7” OR “IL-8” OR “IL-9” OR "IL1" OR "IL2" OR "IL3" OR "IL4" OR "IL5" OR "IL6" OR "IL7" OR "IL8" OR "IL9" OR "IL10" OR "IL11" OR "IL12" OR "IL13" OR "IL14" OR "IL15" OR "IL16" OR "IL17" OR "IL18" OR "IL19" OR "IL20" OR "IL21" OR "IL22" OR "IL23" OR "IL24" OR "IL25" OR "IL26" OR "IL27" OR "IL28" OR "IL29" OR "IL30" OR "IL31" OR "IL32" OR "IL33" OR "IL34" OR "IL35" OR "IL36" OR "IL37" OR "IL38" OR "IL39" OR "IL40" OR “IgG” OR “IgM” OR “IgA” OR “IgE” OR “IgD” OR “lymphocyte*” OR “white blood cell*” OR “WBC” OR “leukocyte*” OR “B-cell*” OR “T-cell*” OR “T-helper” OR “th17“ OR “th 17“ OR “th1” OR “th 1” OR “th2” OR “th 2” OR “neutrophil*” OR “CD4” OR “CD8” OR “basophil*” OR “eosinophil*” OR “granulocyte*” OR “peripheral mononuclear blood cell*” OR “PBMC*” OR “monocyte*” OR “lymphocyte*” OR “interferon*” OR “IFN*” OR “tumor necrosis factor*” OR “TNF*” OR “sTNF*” OR “transforming growth factor*” OR “TGF*” OR “macrophage*” OR “natural killer cell*” OR “NK-cell*” OR “dendritic cell*” OR “mast cell*” OR “plasma cell*” OR “antigen-presenting cell*” OR “APC” OR “lymphoid*” OR “microglia*” OR "neopterin" OR "fibrinogen" OR "E-selectin" OR "serum amyloid A" OR "SAA" OR "erythrocyte sedimentation rate" OR "ESR" OR “platelet count” OR “albumin” OR “high mobility group box 1” OR “HMGB1” OR “HMGB 1” OR “nuclear factor-kappa B” OR “NF-κB” OR “NFκB” OR “NF-kB” OR “NFkB” OR “NF kappa*” OR “NFkappa*” OR “vascular endothelial growth factor” OR “VEGF”)

**Grey literature**

**Clinicaltrials.gov**

Field used: Other terms

("MDD" OR "depression" OR "depressive disorder" OR "depressive disorders")

AND

**("immunological" OR “immune” OR "inflammation" OR “neuroinflammation” OR “neuroimmune” OR “inflammatory” OR “interleukin” OR "interleukins" OR “cytokine” OR “cytokines” OR “chemokine” OR “chemokines” OR “immunoglobulins”** OR “CCL” OR “CXCL” OR “CX3C” OR “C-reactive protein” OR “CRP” OR “lipopolysaccharides” OR “LPS” OR “IL-1” OR “IL-2” OR “IL-3” OR “IL-4” OR “IL-5” OR “IL-6” OR “IL-7” OR “IL-8” OR “IL-9” OR "IL1" OR "IL2" OR "IL3" OR "IL4" OR "IL5" OR "IL6" OR "IL7" OR "IL8" OR "IL9" OR "IL10" OR "IL11" OR "IL12" OR "IL13" OR "IL14" OR "IL15" OR "IL16" OR "IL17" OR "IL18" OR "IL19" OR "IL20" OR "IL21" OR "IL22" OR "IL23" OR "IL24" OR "IL25" OR "IL26" OR "IL27" OR "IL28" OR "IL29" OR "IL30" OR "IL31" OR "IL32" OR "IL33" OR "IL34" OR "IL35" OR "IL36" OR "IL37" OR "IL38" OR "IL39" OR "IL40" OR “IgG” OR “IgM” OR “IgA” OR “IgE” OR “IgD” OR “lymphocytes” OR “white blood cells” OR “WBC” OR “leukocytes” OR “B-cells” OR “T-cells” OR “T-helper” OR “th17“ OR “th 17“ OR “th1” OR “th 1” OR “th2” OR “th 2” OR “neutrophils” OR “CD4” OR “CD8” OR “basophils” OR “eosinophils” OR “granulocytes” OR “peripheral mononuclear blood cells” OR “PBMC” OR “monocytes” OR “lymphocytes” OR “interferon” OR “IFN” OR “IFNγ” OR “IFNg” OR “IFNα” OR “IFNa” OR “IFNβ” OR “IFNb” OR “tumor necrosis factor” OR “TNF” OR “TNFα” OR “TNFa” OR “sTNF” OR “transforming growth factor” OR “TGF” OR “TGFα” OR “TGFa” OR “TGFβ” OR “TGFb” OR “macrophages” OR “natural killer cells” OR “NK-cells” OR “dendritic cells” OR “mast cells” OR “plasma cells” OR “antigen-presenting cells” OR “APC” OR “lymphoids” OR “microglia” OR "neopterin" OR "fibrinogen" OR "E-selectin" OR "serum amyloid A" OR "SAA" OR "erythrocyte sedimentation rate" OR "ESR" OR “platelet count” OR “albumin” OR “high mobility group box 1” OR “HMGB1” OR “HMGB 1” OR “nuclear factor-kappa B” OR “NF-κB” OR “NFκB” OR “NF-kB” OR “NFkB” OR “NF kappa B” OR “Nfkappa B” OR “NF kappaB” OR “NfkappaB” OR “vascular endothelial growth factor” OR “VEGF”)

Filters used

Study results: With results

**ICTRP (**[**https://trialsearch.who.int/**](https://trialsearch.who.int/)**)**

("MDD" OR "depression" OR "depressive disorder*")

AND

("immun*" OR “neuroimmun*” OR “neuroinflamma*” OR "inflamma*" OR "interleukin*" OR “cytokine*” OR “chemokine*” OR “CCL” OR “CXCL” OR “CX3C*” OR “C-reactive protein” OR “CRP” OR “lipopolysaccharide*” OR “LPS” OR “IL-1*” OR “IL-2*” OR “IL-3*” OR “IL-4*” OR “IL-5” OR “IL-6” OR “IL-7” OR “IL-8” OR “IL-9” OR "IL1" OR "IL2" OR "IL3" OR "IL4" OR "IL5" OR "IL6" OR "IL7" OR "IL8" OR "IL9" OR "IL10" OR "IL11" OR "IL12" OR "IL13" OR "IL14" OR "IL15" OR "IL16" OR "IL17" OR "IL18" OR "IL19" OR "IL20" OR "IL21" OR "IL22" OR "IL23" OR "IL24" OR "IL25" OR "IL26" OR "IL27" OR "IL28" OR "IL29" OR "IL30" OR "IL31" OR "IL32" OR "IL33" OR "IL34" OR "IL35" OR "IL36" OR "IL37" OR "IL38" OR "IL39" OR "IL40" OR “IgG” OR “IgM” OR “IgA” OR “IgE” OR “IgD” OR “lymphocyte*” OR “white blood cell*” OR “WBC” OR “leukocyte*” OR “B-cell*” OR “T-cell*” OR “T-helper” OR “th17“ OR “th 17“ OR “th1” OR “th 1” OR “th2” OR “th 2” OR “neutrophil*” OR “CD4” OR “CD8” OR “basophil*” OR “eosinophil*” OR “granulocyte*” OR “peripheral mononuclear blood cell*” OR “PBMC*” OR “monocyte*” OR “lymphocyte*” OR “interferon*” OR “IFN*” OR “tumor necrosis factor*” OR “TNF*” OR “transforming growth factor*” OR “TGF*” OR “macrophage*” OR “natural killer cell*” OR “NK-cell*” OR “dendritic cell*” OR “mast cell*” OR “plasma cell*” OR “antigen-presenting cell*” OR “APC” OR “lymphoid*” OR “microglia*” OR "neopterin" OR "fibrinogen" OR "E-selectin" OR "serum amyloid A" OR "SAA" OR "erythrocyte sedimentation rate" OR "ESR" OR “platelet count” OR “albumin” OR “high mobility group box 1” OR “HMGB1” OR “HMGB 1” OR “nuclear factor-kappa B” OR “NF-κB” OR “NFκB” OR “NF-kB” OR “NFkB” OR “NF kappa*” OR “NFkappa*” OR “vascular endothelial growth factor” OR “VEGF”)

Filters used

With results only

**Europe PubMed Central (including MedrXiv, BiorXiv)**

(

("MDD" OR "depression" OR "depressive disorder" OR "depressive disorders")

AND

("autonomous" OR "autonomic" OR "sympathetic" OR "parasympathetic" OR ANS OR PNS OR SNS OR "heartbeat" OR "heart beat" OR "heart rate" OR HRV OR "catecholamine" OR “catecholamines" OR "epinephrine" OR "norepinephrine" OR "acetylcholine" OR "sympathetic–adrenal–medullary axis" OR SAM OR "RR variability" OR "stress hormone" OR "stress hormones" OR "vagus" OR "vagal" OR "blood pressure" OR "fight-or-flight" OR "respiratory sinus arrhythmia" OR "RSA" OR "sympathoadrenal" OR "sympatho adrenal" OR "cholinergic" OR "cranial nerve" OR "pulse pressure" OR "polyvagal")

AND

**(“immunological” OR “immune” OR "inflammation" OR “neuroinflammation” OR “neuroimmune” OR “inflammatory” OR “interleukin” OR "interleukins" OR “cytokine” OR “cytokines” OR “chemokine” OR “chemokines” OR “immunoglobulins”** OR “CCL” OR “CXCL” OR “CX3C” OR “C-reactive protein” OR “CRP” OR “lipopolysaccharides” OR “LPS” OR “IL-1” OR “IL-2” OR “IL-3” OR “IL-4” OR “IL-5” OR “IL-6” OR “IL-7” OR “IL-8” OR “IL-9” OR "IL1" OR "IL2" OR "IL3" OR "IL4" OR "IL5" OR "IL6" OR "IL7" OR "IL8" OR "IL9" OR "IL10" OR "IL11" OR "IL12" OR "IL13" OR "IL14" OR "IL15" OR "IL16" OR "IL17" OR "IL18" OR "IL19" OR "IL20" OR "IL21" OR "IL22" OR "IL23" OR "IL24" OR "IL25" OR "IL26" OR "IL27" OR "IL28" OR "IL29" OR "IL30" OR "IL31" OR "IL32" OR "IL33" OR "IL34" OR "IL35" OR "IL36" OR "IL37" OR "IL38" OR "IL39" OR "IL40" OR “IgG” OR “IgM” OR “IgA” OR “IgE” OR “IgD” OR “lymphocytes” OR “white blood cells” OR “WBC” OR “leukocytes” OR “B-cells” OR “T-cells” OR “T-helper” OR “th17“ OR “th 17“ OR “th1” OR “th 1” OR “th2” OR “th 2” OR “neutrophils” OR “CD4” OR “CD8” OR “basophils” OR “eosinophils” OR “granulocytes” OR “peripheral mononuclear blood cells” OR “PBMC” OR “monocytes” OR “lymphocytes” OR “interferon” OR “IFN” OR “IFNγ” OR “IFNα” OR “IFNβ” OR “tumor necrosis factor” OR “TNF” OR “TNFα” OR “sTNF” OR “transforming growth factor” OR “TGF” OR “TGFα” OR “TGFβ” OR “macrophages” OR “natural killer cells” OR “NK-cells” OR “dendritic cells” OR “mast cells” OR “plasma cells” OR “antigen-presenting cells” OR “APC” OR “lymphoids” OR “microglia” OR "neopterin" OR "fibrinogen" OR "E-selectin" OR "serum amyloid A" OR "SAA" OR "erythrocyte sedimentation rate" OR "ESR" OR “platelet count” OR “albumin” OR “high mobility group box 1” OR “HMGB1” OR “HMGB 1” OR “nuclear factor-kappa B” OR “NF-κB” OR “NFκB” OR “NF-kB” OR “NFkB” OR “NF kappa B” OR “Nfkappa B” OR “NF kappaB” OR “NfkappaB” OR “vascular endothelial growth factor” OR “VEGF”)

) AND (SRC:PPR)

**SEARCH STRING PART 1:**

**Europe PubMed Central (MedRXiv, BioRXiv)**

(

("MDD" OR "depression" OR "depressive disorder" OR "depressive disorders")

AND

("autonomous" OR "autonomic" OR "sympathetic" OR "parasympathetic" OR ANS OR PNS OR SNS OR "heartbeat" OR "heart beat" OR "heart rate" OR HRV OR "catecholamine" OR “catecholamines" OR "epinephrine" OR "norepinephrine" OR "acetylcholine" OR "sympathetic–adrenal–medullary axis" OR SAM OR "RR variability" OR "stress hormone" OR "stress hormones" OR "vagus" OR "vagal" OR "blood pressure" OR "fight-or-flight" OR "respiratory sinus arrhythmia" OR "RSA" OR "sympathoadrenal" OR "sympatho adrenal" OR "cholinergic" OR "cranial nerve" OR "pulse pressure" OR "polyvagal")

AND

(“immunological” OR “immune” OR "inflammation" OR “neuroinflammation” OR “neuroimmune” OR “inflammatory” OR “interleukin” OR "interleukins" OR “cytokine” OR “cytokines” OR “chemokine” OR “chemokines” OR “immunoglobulins” OR “CCL” OR “CXCL” OR “CX3C” OR “C-reactive protein” OR “CRP” OR “lipopolysaccharides” OR “LPS” OR “IL-1” OR “IL-2” OR “IL-3” OR “IL-4” OR “IL-5” OR “IL-6” OR “IL-7” OR “IL-8” OR “IL-9” OR "IL1" OR "IL2" OR "IL3" OR "IL4" OR "IL5" OR "IL6" OR "IL7" OR "IL8" OR "IL9" OR "IL10" OR "IL11" OR "IL12" OR "IL13" OR "IL14" OR "IL15" OR "IL16" OR "IL17" OR "IL18" OR "IL19" OR "IL20" OR "IL21" OR "IL22" OR "IL23" OR "IL24" OR "IL25" OR "IL26" OR "IL27" OR "IL28" OR "IL29" OR "IL30" OR "IL31" OR "IL32" OR "IL33" OR "IL34" OR "IL35" OR "IL36" OR "IL37" OR "IL38" OR "IL39" OR "IL40" OR “IgG” OR “IgM” OR “IgA” OR “IgE” OR “IgD”)

) AND (SRC:PPR)

**SEARCH STRING PART 2:**

(

("MDD" OR "depression" OR "depressive disorder" OR "depressive disorders")

AND

("autonomous" OR "autonomic" OR "sympathetic" OR "parasympathetic" OR ANS OR PNS OR SNS OR "heartbeat" OR "heart beat" OR "heart rate" OR HRV OR "catecholamine" OR “catecholamines" OR "epinephrine" OR "norepinephrine" OR "acetylcholine" OR "sympathetic–adrenal–medullary axis" OR SAM OR "RR variability" OR "stress hormone" OR "stress hormones" OR "vagus" OR "vagal" OR "blood pressure" OR "fight-or-flight" OR "respiratory sinus arrhythmia" OR "RSA" OR "sympathoadrenal" OR "sympatho adrenal" OR "cholinergic" OR "cranial nerve" OR "pulse pressure" OR "polyvagal")

AND

(“lymphocytes” OR “white blood cells” OR “WBC” OR “leukocytes” OR “B-cells” OR “T-cells” OR “T-helper” OR “th17“ OR “th 17“ OR “th1” OR “th 1” OR “th2” OR “th 2” OR “neutrophils” OR “CD4” OR “CD8” OR “basophils” OR “eosinophils” OR “granulocytes” OR “peripheral mononuclear blood cells” OR “PBMC” OR “monocytes” OR “lymphocytes” OR “interferon” OR “IFN” OR “IFNγ” OR “IFNα” OR “IFNβ” OR “tumor necrosis factor” OR “TNF” OR “TNFα” OR “sTNF” OR “transforming growth factor” OR “TGF” OR “TGFα” OR “TGFβ”)

) AND (SRC:PPR)

**SEARCH STRING PART 3**

(

("MDD" OR "depression" OR "depressive disorder" OR "depressive disorders")

AND

("autonomous" OR "autonomic" OR "sympathetic" OR "parasympathetic" OR ANS OR PNS OR SNS OR "heartbeat" OR "heart beat" OR "heart rate" OR HRV OR "catecholamine" OR “catecholamines" OR "epinephrine" OR "norepinephrine" OR "acetylcholine" OR "sympathetic–adrenal–medullary axis" OR SAM OR "RR variability" OR "stress hormone" OR "stress hormones" OR "vagus" OR "vagal" OR "blood pressure" OR "fight-or-flight" OR "respiratory sinus arrhythmia" OR "RSA" OR "sympathoadrenal" OR "sympatho adrenal" OR "cholinergic" OR "cranial nerve" OR "pulse pressure" OR "polyvagal")

AND

(“macrophages” OR “natural killer cells” OR “NK-cells” OR “dendritic cells” OR “mast cells” OR “plasma cells” OR “antigen-presenting cells” OR “APC” OR “lymphoids” OR “microglia” OR "neopterin" OR "fibrinogen" OR "E-selectin" OR "serum amyloid A" OR "SAA" OR "erythrocyte sedimentation rate" OR "ESR" OR “platelet count” OR “albumin” OR “high mobility group box 1” OR “HMGB1” OR “HMGB 1” OR “nuclear factor-kappa B” OR “NF-κB” OR “NFκB” OR “NF-kB” OR “NFkB” OR “NF kappa B” OR “Nfkappa B” OR “NF kappaB” OR “NfkappaB” OR “vascular endothelial growth factor” OR “VEGF”)

) AND (SRC:PPR)
