# Supplementary material for: The interaction between the immune system and the autonomic nervous system in the pathophysiology of major depressive disorder: a scoping review
Source: Brain Behav Immun Health. 2026 Aug 22;56:101334. doi: 10.1016/j.bbih.2026.101334 (PMC13527485; doi:10.1016/j.bbih.2026.101334)
Supplement: Supplementary file 2 [file mmc2.docx]

***Supplementary table S1:*** *Overview of HRV indices across papers and their physiological interpretation. Adapted under a CC BY 4.0 license from previous literature (1, 2).*

| **Domain** | **Variable** | **Unit** | **Description** | **Interpretation** |
| --- | --- | --- | --- | --- |
| Time-domain | RMSSD | ms | Root mean square of successive NN interval differences | Vagal tone |
|  | SDNN | ms | Standard deviation of NN intervals | Overall level of HRV |
|  | SDANN | ms | Standard deviation of average NN intervals | Long-term HRV components |
|  | SDNNI | ms | Mean of the standard deviations of NN intervals | Autonomic regulation within 5-min periods |
|  | pRR50 / pNN50 | % | Percentage of successive NN intervals differing by >50 ms | Vagal tone |
|  | Mean RR interval | ms | Average time between consecutive normal heartbeats | Inverse of HR |
|  | SD RR interval | ms | Standard deviation of RR intervals | Overall variability in RR intervals |
|  | HRV triangular index (TRIIDX) |  | Integral of NN interval histogram divided by its height | Overall autonomic variability |
| Frequency-domain | VLF | ms² | Very low frequency power (≤0.04 Hz) | Slow autonomic regulatory processes |
|  | LF | ms² | Low frequency power (0.04–0.15 Hz) | Combined regulatory effects of both SNS and PNS |
|  | HF | ms² | High frequency power (0.15–0.40 Hz) | Vagal tone |
|  | Total Power (TP) | ms² | Total variance in HRV spectrum | Overall autonomic variability |
|  | LF/HF ratio | % | Ratio of LF-to-HF power | Sympathovagal balance |
|  | HF power | n.u. | Relative power of the high-frequency band (0.15–0.4 Hz) in normal units | Relative parasympathetic contribution to HRV |
|  | LF power | n.u. | Relative power of the low-frequency band (0.04–0.15 Hz) in normal units | Baroreflex- mediated autonomic modulation |
|  | HF power | % | Relative power of the high-frequency band (0.15–0.4 Hz) | Relative contribution of parasympathetic modulation |
|  | lnVLF | ln(ms²) | Natural logarithm of very low frequency power |  |
|  | lnLF | ln(ms²) | Natural logarithm of low frequency power |  |
|  | lnHF | ln(ms²) | Natural logarithm of high frequency power |  |
|  | LnTP | ln(ms²) | Natural logarithm of total power |  |
| Respiratory | RSA | ms or ln(ms²) | Respiratory sinus arrhythmia | Vagal cardiac control linked to respiration |
|  | Average respiration interval | ms | Mean interval between respiratory cycles | Respiratory rate influencing cardiovagal modulation and HRV amplitude |

*Note. NN intervals = normal-to-normal interbeat intervals (intervals between successive normal heartbeats); RR intervals = intervals between successive R waves; n.u. = normalized units*

**Supplementary references:**

1.	Laborde S, Mosley E, Thayer JF. Heart Rate Variability and Cardiac Vagal Tone in Psychophysiological Research - Recommendations for Experiment Planning, Data Analysis, and Data Reporting. Front Psychol. 2017;8:213.

2.	Pham T, Lau ZJ, Chen SHA, Makowski D. Heart Rate Variability in Psychology: A Review of HRV Indices and an Analysis Tutorial. Sensors (Basel). 2021;21(12).
